# Supplementary material for: Hybrid Mass Spectrometry Approaches to Determine How L-Histidine Feedback Regulates the Enzyzme MtATP-Phosphoribosyltransferase
Source: Structure. 2017 May 2;25(5):730–738.e4. doi: 10.1016/j.str.2017.03.005 (PMC5415358; doi:10.1016/j.str.2017.03.005)
Supplement: Document S1. Figures S1–S6 and Tables S1–S3 [file mmc1.pdf]

Structure, Volume *25*

Supplemental Information

Hybrid Mass Spectrometry Approaches to Determine How L-Histidine Feedback Regulates the Enzyzme MtATP-Phosphoribosyltransferase

Kamila J. Pacholarz, Rebecca J. Burnley, Thomas A. Jowitt, Victoria Ordsmith, João Pedro Pisco, Massimiliano Porrini, Gérald Larrouy-Maumus, Rachel A. Garlish, Richard J. Taylor, Luiz Pedro Sório de Carvalho, and Perdita E. Barran

Supplemental Information

Hybrid Mass Spectrometry Approaches to Determine how L-Histidine Feedback Regulates the Enzyme MtATP-Phosphoribosyltransferase

Kamila J. Pacholarz[1,2], Rebecca J. Burnley[3], Thomas A. Jowitt[4], Victoria Ordsmith [2], João Pedro Pisco[5], Massimiliano Porrini[6], Gérald Larrouy-Maumus[5,$], Rachel A. Garlish[3], Richard J. Taylor[3], Luiz Pedro Sório de Carvalho[5*], Perdita E. Barran[1 *]

[1] Michael Barber Centre for Collaborative Mass Spectrometry, Manchester Institute of Biotechnology, 131 Princess Street, M1 7DN & School of Chemistry, University of Manchester, Oxford Road, M13 9PL, Manchester, UK
[2]School of Chemistry, University of Edinburgh, David Brewster Road, EH9 3FJ, Edinburgh, UK
[3] Physical and Analytical Sciences Group, UCB, 216 Bath Road, SL1 4EN, Slough, UK
[4] The Biomolecular Analysis Facility, Faculty of Life Sciences, University of Manchester, Oxford Road, M13 9PT, Manchester, UK
[5] Mycobacterial Metabolism and Antibiotic Research Laboratory, The Francis Crick Institute, 1 Midland Road, NW1 1AT, London, UK
[6] ARNA Laboratory, Inserm U1212/CNRS UMR 5320/University of Bordeaux, France
[1] Lead Contact, perdita.barran@manchester.ac.uk

Supporting Data

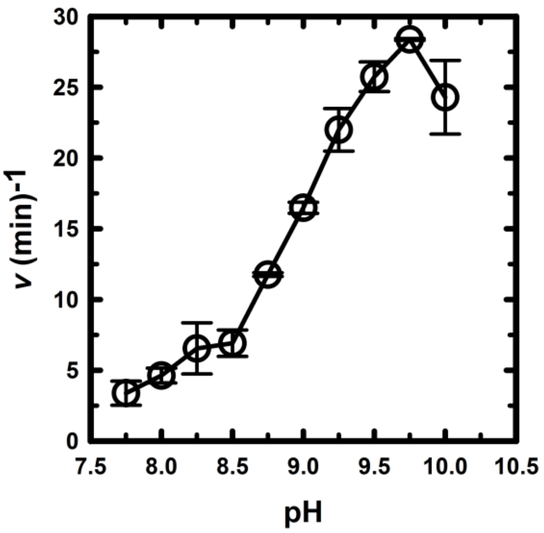

Figure S1. Related to Figure 1. MtATP-PRT activity pH dependence. Below pH 7.75, despite very slow, MtATP-PRT is still active.

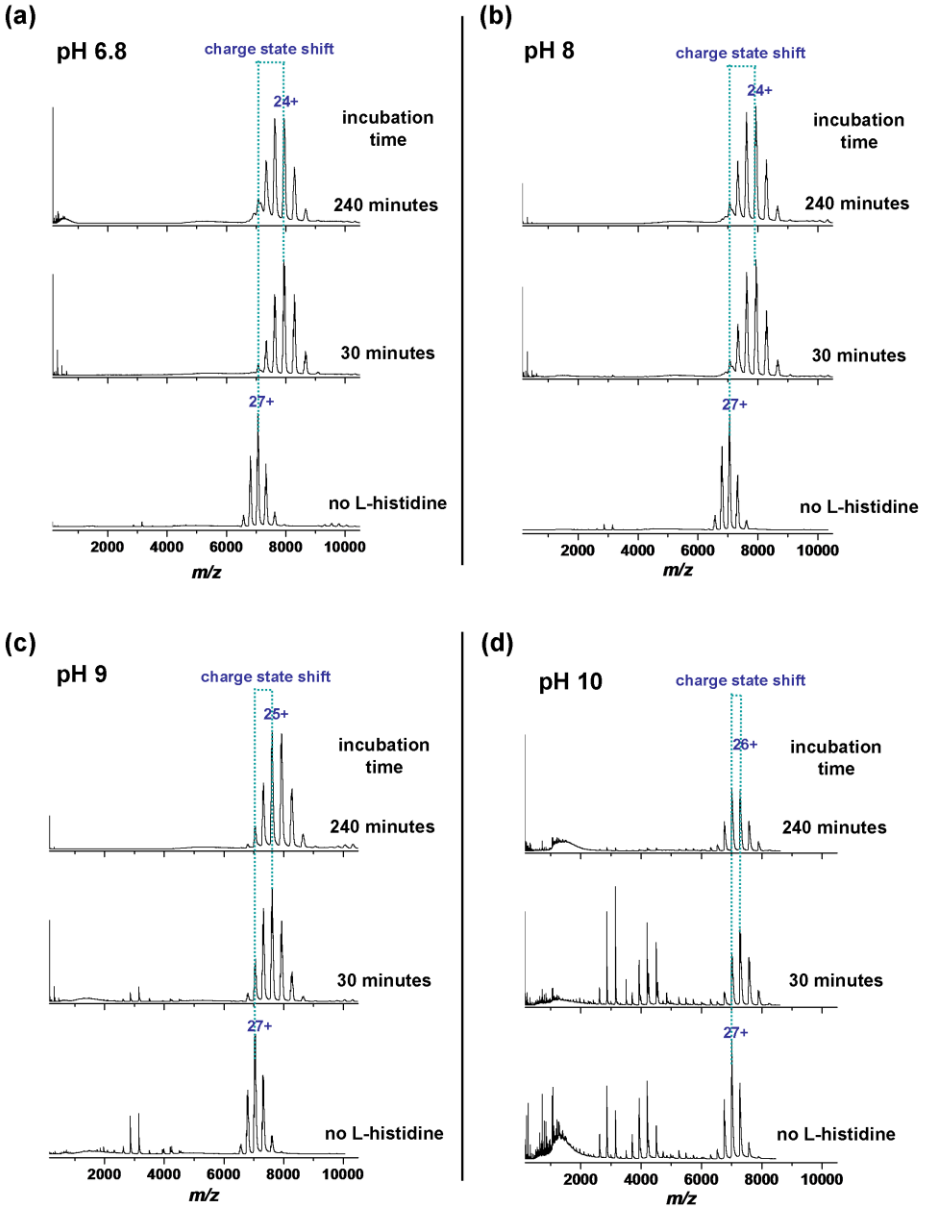

Figure S2. Related to Figure 2. nESI mass spectra of 20 µM apo MtATP-PRT (hexamer concentration) and MtATP-PRT incubated with 12 equivalents of L-histidine for 30 minutes and 240 minutes in 100 mM ammonium acetate at (a) pH 6.8, (b) pH 8, (c) pH 9 and (d) pH 10, acquired on Ultima API US Q-ToF mass spectrometer (at cone voltage 268 V). Efforts to shift charge state distribution (CSD) to a higher *m/z* range by addition of charge reducing agents such as TEAA and EDDA (*5*) did not improve resolution sufficiently to determine the binding stoichiometry.

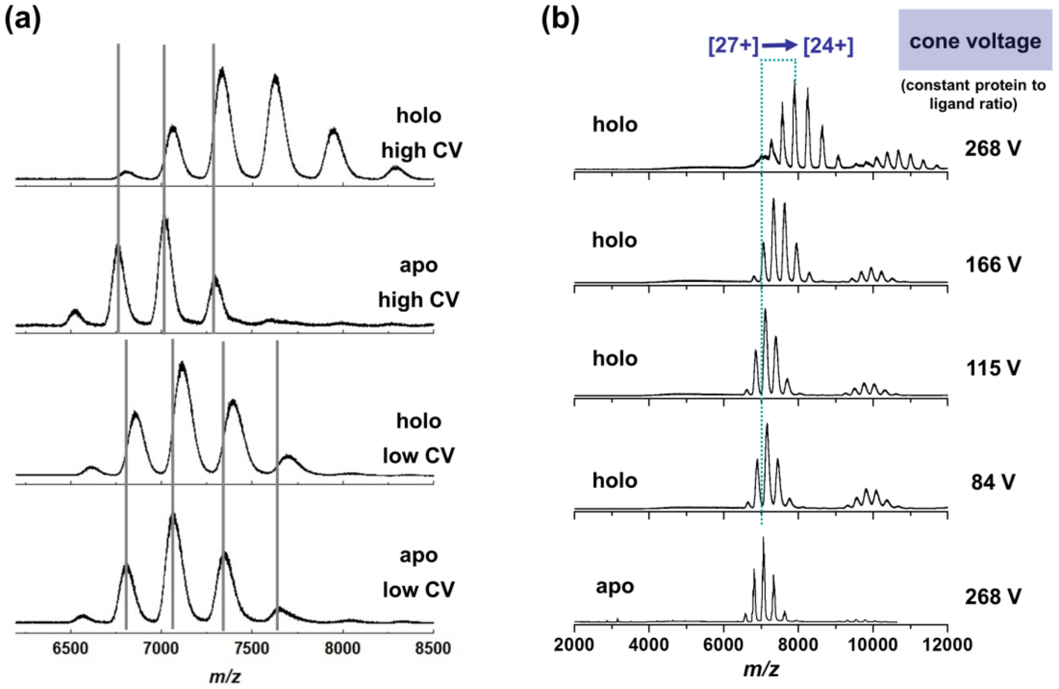

Figure S3. Related to Figure 3 . (a) Mass spectra of MtATP-PRT in the presence and absence of 12 equivalents of L-histidine (denoted as 'holo'; 1 MtATP-PRT hexamer (H):12 ligand (L)) in 100 mM ammonium acetate at pH 6.8 at 115 V (low CV) and 166 V cone voltage (high CV), showing the mass shift associated with ligand binding. Moreover, a shift in the charge state envelope is noted at higher CV. Grey lines are used to help visualize the mass shift (based on apex of 'no ligand' peaks). (b) Dissociation of L-histidine upon increased cone voltage; 1 equivalent of MtATP-PRT incubated with 12 equivalents of L-histidine in 100 mM ammonium acetate pH 6.8 acquired at various cone voltages ranging from 84 – 268 V on Ultima API US Q-ToF mass spectrometer.

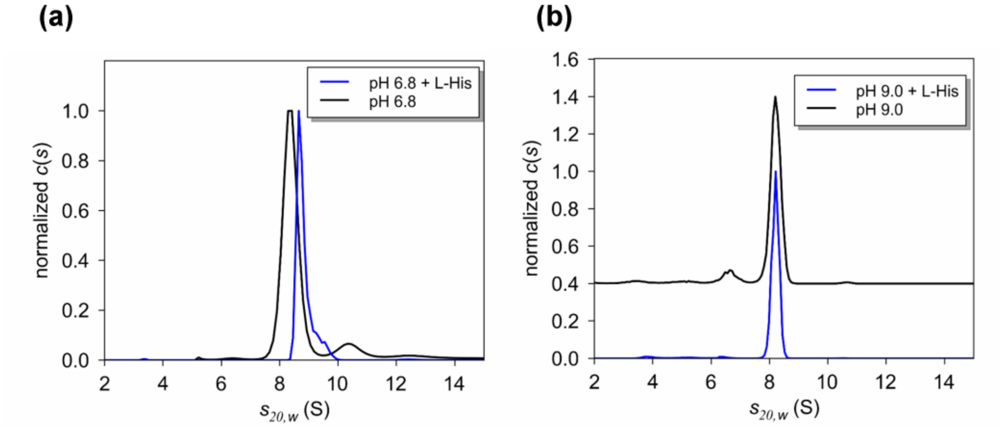

Figure S4. Related to Figure 2b. Sedimentation coefficient distributions ($s_{20,w}$) of MtATP-PRT in 100 mM ammonium acetate at pH 6.8 (a) and pH 9 (b), in presence (blue trace) and in absence of L-histidine (black trace). More compact proteins experience less hydrodynamic friction, and thus have larger sedimentation coefficient.

Table S1. Related to Figure 2b. Results from the bead modelling of the apo and holo forms of MtATP-PRT compared to the data from AUC measurements: sedimentation coefficient (S), diffusion coefficient (D), hydrodynamic radius (R_h), radius of gyration (R_g) and total bead surface area (A^b).

	Experimental		Bead model	
	**Apo**	**Holo**	**Apo**	**Holo**
**S**	8.22	8.58	8.38	8.54
**D (cm/sec^2)**	-	-	4.50	4.54
**R_h (nm)**	5.49	5.25	4.76	4.72
**R_g (nm)**	-	-	3.75	3.75
**f/f_0**	1.37	1.28	1.27	1.26
**A^b(nm^2)**	-	-	655	641

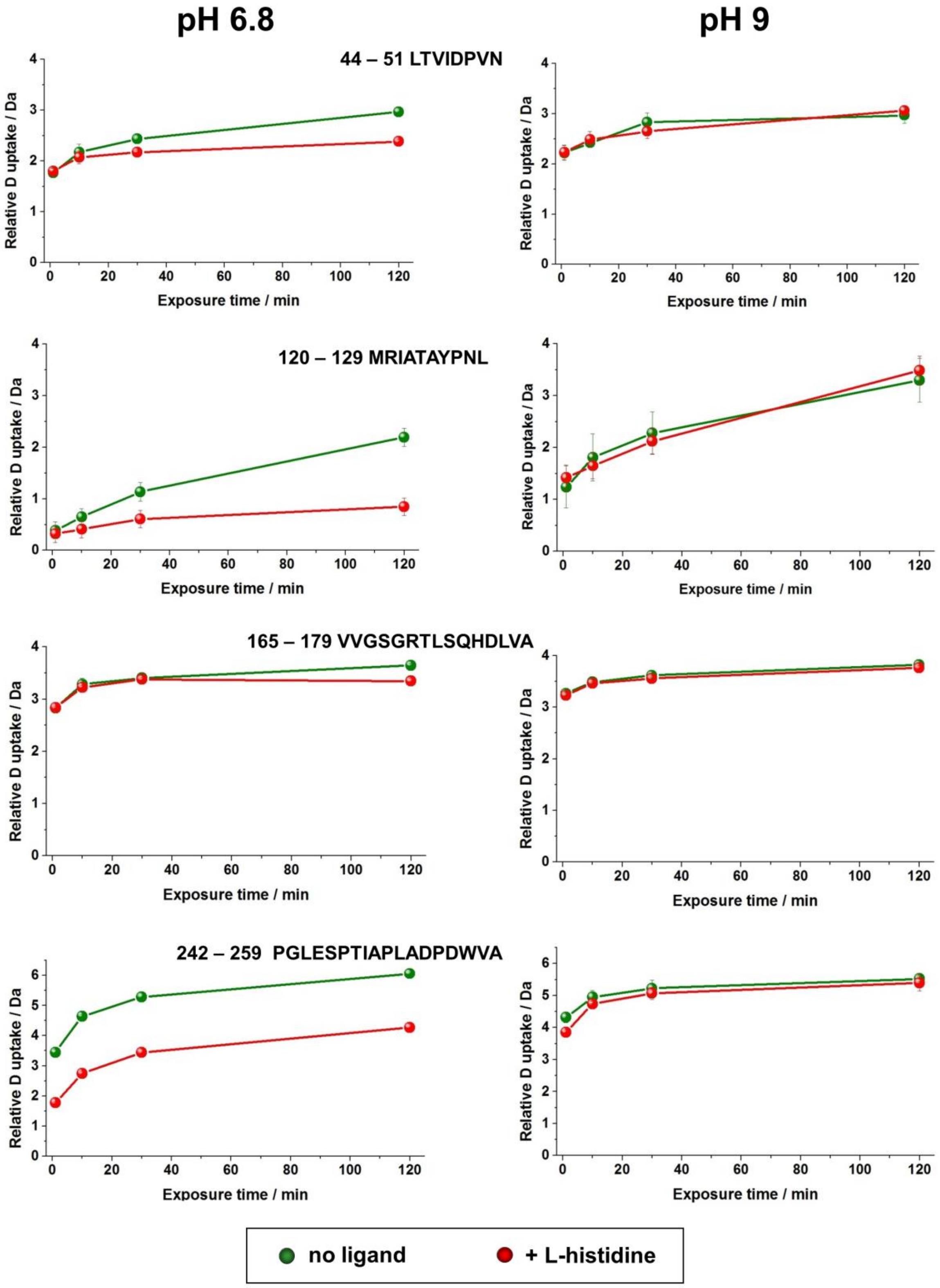

Figure S5. Related to Figure 4. Example deuterium uptake curves of four chosen peptides of MtATP-PRT in presence (red) and in absence (green) of L-histidine at pH 6.8 (left) and pH 9 (right). Deuterium uptake was determined at four exposure time points: 1 minute, 10 minutes, 30 minutes and 120 minutes. D uptake data for all peptides identified is provided below.

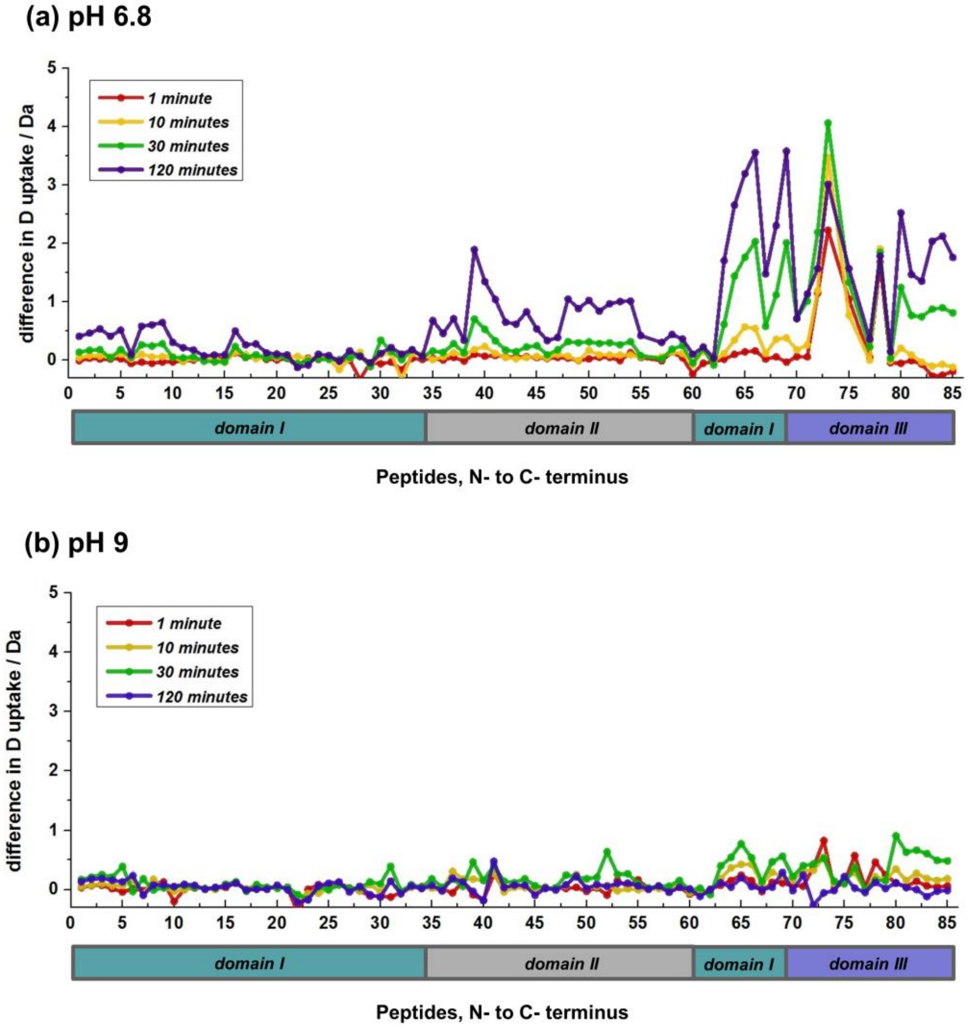

Figure S6. Related to Figure 4. Difference in the absolute D uptake between L-histidine free and L-histidine bound MtATP-PRT at pH 6.8 (a) and pH 9 (b), measured at four deuterium exposure time points: 1 minute (red), 10 minutes (yellow), 30 minutes (green) and 120 minutes (blue); providing information about regions of ligand binding and conformational changes.

Table S2. Related to Figure 4. List of MtATP-PRT residues with altered D uptake due to L-histidine binding determined via HDX experiments; along with list of MtATP-PRT involved in binding with L-histidine, AMP and residues predicted to interact with ATP and PRPP.

**HDX DATA**		
**Residues**	**Change in D uptake**	**Peptide number**
L44-F55	long time scale: 120 min	7-9
L117-L129	intermediate time scale: 30 min	39-41
V130-E151	intermediate and long time scale: 30-120 min	44-45
I152-D164	intermediate and long time scale: 30-120 min	48-53
V210-L234	intermediate and long time scale: 30-120 min (significant)	63-71
D226-A259	instant within 1 minute	72, 73, 75, 78
Y227-K235	intermediate and long time scale: 30-120 min	74
V258-L285	intermediate and long time scale: 30-120 min	80-85

**X-RAY CRYSTALLOGRAPHY DATA (*6*)**	
**Residues (HDX numbering)**	**Type of interaction**
D228, L244, S246, T248, L263, A283	L-histidine
D40, D43,D80, S100, Y126,D164-T171	AMP
K19, K42, R59, R170	ATP and PRPP (predicted)

Table S3a. Related to Figure 4. List of MtATP-PRT peptides identified after digestion with pepsin.

peptide #	Start residue	End residue	Maximum D uptake	Sequence
1	12	27	13	LRVAVPNKGALSEPAT
2	12	28	14	LRVAVPNKGALSEPATE
3	12	30	16	LRVAVPNKGALSEPATEIL
4	13	28	13	RVAVPNKGALSEPATE
5	13	30	15	RVAVPNKGALSEPATEIL
6	29	35	6	ILAEAGY
7	44	51	6	LTVIDPVN
8	44	52	7	LTVIDPVNN
9	44	55	10	LTVIDPVNNVEF
10	45	55	9	TVIDPVNNVEF
11	46	55	8	VIDPVNNVEF
12	47	55	7	IDPVNNVEF
13	56	62	5	FFLRPKD
14	56	64	7	FFLRPKDIA
15	57	65	7	FLRPKDIAI
16	63	71	8	IAIYVGSGE
17	63	73	10	IAIYVGSGELD
18	63	74	11	IAIYVGSGELDF
19	65	74	9	IYVGSGELDF
20	66	73	7	YVGSGELD
21	66	74	8	YVGSGELDF
22	66	80	14	YVGSGELDFGITGRD
23	67	80	13	VGSGELDFGITGRD
24	72	80	8	LDFGITGRD
25	73	79	6	DFGITGR
26	73	80	7	DFGITGRD
27	81	88	7	LVCDSGAQ
28	81	91	10	LVCDSGAQVRE
29	84	93	9	DSGAQVRERL
30	89	102	13	VRERLALGFGSSSF
31	92	102	10	RLALGFGSSSF
32	94	101	7	ALGFGSSS
33	94	102	8	ALGFGSSSF
34	96	102	6	GFGSSSF
35	102	111	8	FRYAAPAGRN
36	103	111	7	RYAAPAGRN
37	103	117	13	RYAAPAGRNWTTADL
38	112	119	7	WTTADLAG
39	117	129	11	LAGMRIATAYPNL
40	120	129	8	MRIATAYPNL
41	121	129	7	RIATAYPNL
42	130	140	10	VRKDLATKGIE
43	130	141	11	VRKDLATKGIEA
44	130	142	12	VRKDLATKGIEAT
45	142	151	9	TVIRLDGAVE
46	143	149	6	VIRLDGA
47	143	151	8	VIRLDGAVE

peptide #	Start residue	End residue	Maximum D uptake	Sequence
48	152	159	7	ISVQLGVA
49	152	160	8	ISVQLGVAD
50	152	161	9	ISVQLGVADA
51	154	161	7	VQLGVADA
52	157	163	6	GVADAIA
53	157	164	7	GVADAIAD
54	160	179	19	DAIADVVGSGRTLSQHDLVA
55	162	179	17	IADVVGSGRTLSQHDLVA
56	164	179	15	DVVGSGRTLSQHDLVA
57	165	179	14	VVGSGRTLSQHDLVA
58	180	186	5	FGEPLCD
59	180	188	7	FGEPLCDSE
60	190	209	19	VLIERAGTDGQDQTEARDQL
61	191	199	8	LIERAGTDG
62	192	209	17	IERAGTDGQDQTEARDQL
63	210	218	8	VARVQGVVF
64	210	221	11	VARVQGVVFGQQ
65	210	222	12	VARVQGVVFGQQY
66	210	223	13	VARVQGVVFGQQYL
67	212	218	6	RVQGVVF
68	212	221	9	RVQGVVFGQQ
69	212	223	11	RVQGVVFGQQYL
70	225	233	7	LDYDCPRSA
71	225	234	8	LDYDCPRSAL
72	226	246	18	DYDCPRSALKKATAITPGLES
73	226	259	28	DYDCPRSALKKATAITPGLESPTIAPLADPDWVA
74	227	234	6	YDCPRSAL
75	235	246	10	KKATAITPGLES
76	235	259	14	KKATAITPGLESPTIA
77	237	245	7	ATAITPGLE
78	242	259	14	PGLESPTIAPLADPDWVA
79	249	257	6	IAPLADPDW
80	258	273	14	VAIRALVPRRDVNGIM
81	260	273	12	IRALVPRRDVNGIM
82	260	275	14	IRALVPRRDVNGIMDE
83	276	285	9	LAAIGAKAIL
84	277	285	8	AAIGAKAIL
85	279	285	6	IGAKAIL

Table S3b. Related to Figure 4. Average relative D uptake at 1 minute, 10 minutes, 30 minutes and 120 minutes of exposure along with standard deviation values (SD) for apo MtATP-PRT at pH 6.8.

pH 6.8	D UPTAKE /Da							
peptide #	1 min	SD	10 min	SD	30 min	SD	120 min	SD
1	3.04	0.04	3.04	0.05	3.38	0.08	4.08	0.09
2	3.06	0.04	3.04	0.03	3.38	0.05	4.07	0.10
3	2.86	0.05	2.84	0.04	3.12	0.05	3.79	0.08
4	3.05	0.03	3.02	0.05	3.30	0.06	4.01	0.10
5	2.98	0.08	2.99	0.07	3.27	0.08	3.95	0.14
6	0.30	0.04	0.33	0.05	0.46	0.04	0.75	0.04
7	1.77	0.09	2.17	0.16	2.43	0.09	2.96	0.09
8	1.90	0.06	2.48	0.04	2.86	0.06	3.39	0.08
9	1.85	0.03	2.29	0.05	2.69	0.04	3.27	0.09
10	1.73	0.12	2.08	0.05	2.25	0.09	2.43	0.24
11	1.19	0.03	1.50	0.06	1.70	0.04	1.89	0.06
12	1.10	0.08	1.42	0.05	1.54	0.05	1.69	0.08
13	0.91	0.02	1.04	0.02	1.21	0.02	1.39	0.02
14	1.18	0.11	1.62	0.05	1.86	0.03	2.12	0.03
15	1.06	0.10	1.62	0.11	1.94	0.09	2.21	0.11
16	1.16	0.10	1.51	0.06	1.87	0.07	2.37	0.06
17	0.61	0.03	0.93	0.03	1.18	0.07	1.73	0.04
18	0.48	0.06	0.69	0.05	1.02	0.07	1.57	0.13
19	0.62	0.04	0.91	0.03	1.27	0.03	1.74	0.04
20	0.53	0.01	0.84	0.02	1.20	0.03	1.65	0.07
21	0.68	0.03	1.05	0.02	1.45	0.03	2.02	0.06
22	0.94	0.12	1.69	0.12	2.12	0.02	2.54	0.15
23	1.13	0.09	1.63	0.10	2.05	0.03	2.34	0.04
24	0.92	0.07	1.16	0.06	1.23	0.06	1.32	0.15
25	0.72	0.02	0.92	0.01	1.00	0.01	1.06	0.01
26	0.74	0.11	0.86	0.07	1.04	0.10	1.03	0.06
27	1.96	0.03	1.96	0.03	2.05	0.07	2.23	0.04
28	3.00	0.04	3.54	0.12	3.72	0.15	3.72	0.08
29	2.51	0.14	2.70	0.16	2.83	0.12	2.91	0.18
30	2.28	0.20	2.54	0.13	3.02	0.28	3.40	0.19
31	1.86	0.03	2.16	0.01	2.50	0.06	3.05	0.03
32	1.97	0.03	2.04	0.07	2.17	0.02	2.57	0.04
33	1.24	0.03	1.49	0.04	1.86	0.06	2.39	0.07
34	1.60	0.01	1.74	0.01	1.86	0.02	2.00	0.02
35	2.25	0.06	2.41	0.06	2.56	0.05	3.05	0.13
36	2.31	0.04	2.46	0.01	2.56	0.03	2.87	0.02
37	3.69	0.04	4.02	0.02	4.42	0.05	5.17	0.08
38	1.17	0.05	1.30	0.02	1.62	0.07	2.15	0.04
39	0.55	0.08	0.88	0.06	1.70	0.12	3.17	0.08
40	0.39	0.16	0.65	0.16	1.13	0.18	2.19	0.18
41	0.39	0.24	0.59	0.20	0.98	0.21	1.92	0.20
42	1.35	0.04	2.08	0.04	2.37	0.03	2.88	0.05
43	1.95	0.06	2.79	0.05	3.04	0.05	3.55	0.04
44	2.08	0.15	3.15	0.19	3.54	0.14	4.33	0.10
45	2.17	0.09	2.86	0.08	3.47	0.06	4.27	0.13

peptide #	1 min	SD	10 min	SD	30 min	SD	120 min	SD
46	1.33	0.05	1.56	0.06	1.80	0.08	2.17	0.07
47	2.01	0.04	2.63	0.06	3.12	0.04	3.75	0.02
48	1.37	0.03	1.75	0.03	2.07	0.01	2.91	0.03
49	1.45	0.11	1.83	0.07	2.19	0.06	2.97	0.08
50	1.70	0.17	2.16	0.36	2.43	0.04	3.29	0.10
51	0.70	0.03	1.00	0.03	1.27	0.05	1.86	0.05
52	0.40	0.06	0.38	0.04	0.61	0.07	1.29	0.07
53	0.45	0.08	0.44	0.02	0.68	0.08	1.48	0.12
54	6.01	0.07	6.14	0.07	6.72	0.12	7.97	0.11
55	4.44	0.11	4.84	0.09	5.23	0.10	6.00	0.07
57	2.83	0.04	3.28	0.12	3.40	0.07	3.64	0.03
58	0.71	0.03	0.70	0.05	0.82	0.09	1.28	0.04
59	0.56	0.04	0.66	0.04	0.96	0.05	1.46	0.07
60	6.35	0.18	6.93	0.10	7.17	0.16	7.62	0.15
61	1.16	0.09	1.48	0.12	1.87	0.15	2.40	0.08
62	5.97	0.06	6.40	0.04	6.60	0.13	7.01	0.12
63	0.24	0.02	0.32	0.02	0.86	0.02	2.02	0.02
64	0.58	0.05	0.95	0.07	2.31	0.06	3.89	0.06
65	0.63	0.11	1.19	0.08	2.72	0.03	4.56	0.07
66	0.61	0.04	1.21	0.05	2.93	0.03	4.82	0.15
67	0.15	0.03	0.22	0.02	0.69	0.04	1.66	0.03
68	0.57	0.06	0.99	0.07	1.94	0.07	3.52	0.14
69	0.49	0.07	1.03	0.07	2.83	0.16	4.75	0.08
70	0.72	0.05	1.08	0.10	2.02	0.07	2.63	0.08
71	0.82	0.03	1.26	0.04	2.44	0.03	3.22	0.03
72	4.31	0.31	5.62	0.18	7.56	0.30	8.65	0.27
73	6.10	0.18	9.13	0.17	11.32	0.26	13.03	0.31
74	0.80	0.09	1.17	0.10	2.05	0.09	2.57	0.09
75	2.55	0.04	3.07	0.17	4.11	0.02	4.83	0.17
77	1.48	0.06	1.75	0.02	2.35	0.02	2.91	0.05
78	3.45	0.09	4.65	0.06	5.29	0.17	6.06	0.15
79	1.20	0.03	1.82	0.14	2.07	0.14	2.38	0.07
80	1.49	0.08	1.81	0.09	3.28	0.07	5.24	0.16
81	1.53	0.03	1.77	0.04	2.84	0.03	4.24	0.07
82	1.68	0.13	2.00	0.17	3.29	0.18	4.65	0.18
83	0.53	0.02	1.50	0.03	2.86	0.02	4.32	0.03
84	0.50	0.02	1.38	0.03	2.61	0.03	3.92	0.04
85	0.42	0.04	1.08	0.06	2.23	0.10	3.26	0.16

Table S3c. Related to Figure 4. Average relative D uptake at 1 minute, 10 minutes, 30 minutes and 120 minutes of exposure along with standard deviation values (SD) for MtATP-PRT in presence of L-histidine at pH 6.8.

pH 6.8	D UPTAKE /Da							
peptide #	1 min	SD	10 min	SD	30 min	SD	120 min	SD
1	3.05	0.03	3.00	0.03	3.25	0.03	3.67	0.06
2	3.03	0.04	2.97	0.04	3.22	0.02	3.60	0.05
3	2.84	0.03	2.77	0.02	2.94	0.03	3.25	0.06
4	3.03	0.04	2.97	0.03	3.26	0.03	3.60	0.06
5	2.95	0.08	2.89	0.09	3.09	0.08	3.43	0.10
6	0.36	0.06	0.31	0.06	0.41	0.03	0.65	0.04
7	1.80	0.05	2.07	0.13	2.17	0.04	2.38	0.04
8	1.95	0.05	2.43	0.04	2.61	0.04	2.78	0.04
9	1.89	0.11	2.23	0.05	2.41	0.10	2.62	0.06
10	1.76	0.08	2.02	0.17	2.20	0.05	2.13	0.12
11	1.21	0.02	1.53	0.04	1.66	0.05	1.67	0.02
12	1.10	0.06	1.39	0.06	1.48	0.08	1.51	0.06
13	0.87	0.02	1.03	0.02	1.23	0.02	1.32	0.03
14	1.12	0.05	1.58	0.05	1.89	0.05	2.03	0.05
15	1.06	0.08	1.66	0.14	1.96	0.06	2.12	0.09
16	1.04	0.03	1.37	0.02	1.64	0.04	1.87	0.05
17	0.56	0.04	0.83	0.06	1.13	0.03	1.47	0.02
18	0.42	0.07	0.67	0.09	0.93	0.07	1.29	0.06
19	0.61	0.03	0.86	0.03	1.23	0.03	1.62	0.03
20	0.53	0.02	0.80	0.03	1.13	0.01	1.54	0.03
21	0.65	0.03	1.00	0.02	1.42	0.02	1.93	0.04
22	1.05	0.05	1.63	0.04	2.18	0.11	2.67	0.05
23	1.10	0.11	1.61	0.10	2.06	0.09	2.42	0.05
24	0.90	0.05	1.11	0.06	1.21	0.06	1.21	0.07
25	0.69	0.02	0.89	0.01	0.98	0.01	0.98	0.02
26	0.76	0.11	1.02	0.11	1.03	0.11	1.05	0.12
27	1.96	0.02	1.93	0.03	2.01	0.04	2.07	0.04
28	3.33	0.16	3.40	0.05	3.64	0.01	3.66	0.08
29	2.56	0.18	2.80	0.10	2.94	0.08	2.96	0.15
30	2.35	0.07	2.45	0.15	2.68	0.13	3.28	0.14
31	1.89	0.05	2.04	0.02	2.35	0.05	2.83	0.06
32	2.14	0.14	2.38	0.06	2.17	0.07	2.47	0.03
33	1.20	0.04	1.37	0.03	1.68	0.04	2.21	0.06
34	1.59	0.04	1.64	0.03	1.80	0.02	1.93	0.06
35	2.21	0.06	2.40	0.07	2.40	0.03	2.37	0.05
36	2.31	0.04	2.40	0.03	2.43	0.04	2.41	0.04
37	3.64	0.04	3.89	0.02	4.14	0.02	4.46	0.08
38	1.18	0.05	1.21	0.04	1.49	0.03	1.81	0.04
39	0.45	0.04	0.69	0.05	0.99	0.04	1.28	0.04
40	0.32	0.17	0.41	0.17	0.61	0.17	0.85	0.17
41	0.31	0.28	0.48	0.27	0.64	0.27	0.88	0.28
42	1.30	0.03	2.03	0.03	2.20	0.03	2.23	0.04
43	1.90	0.04	2.76	0.04	2.89	0.05	2.93	0.05
44	2.02	0.10	3.10	0.15	3.32	0.18	3.51	0.22
45	2.13	0.08	2.80	0.07	3.23	0.06	3.73	0.07

peptide #	1 min	SD	10 min	SD	30 min	SD	120 min	SD
46	1.30	0.03	1.52	0.06	1.71	0.09	1.83	0.07
47	1.96	0.02	2.53	0.02	2.94	0.03	3.36	0.03
48	1.33	0.01	1.69	0.01	1.75	0.02	1.86	0.03
49	1.46	0.05	1.84	0.06	1.90	0.03	2.09	0.06
50	1.68	0.31	1.99	0.03	2.11	0.04	2.27	0.06
51	0.65	0.03	0.91	0.01	0.98	0.01	1.02	0.01
52	0.35	0.03	0.29	0.03	0.31	0.03	0.33	0.04
53	0.45	0.02	0.36	0.02	0.42	0.07	0.48	0.05
54	5.88	0.09	6.05	0.04	6.40	0.05	6.96	0.12
55	4.39	0.11	4.78	0.08	5.15	0.07	5.58	0.10
57	2.84	0.03	3.23	0.03	3.38	0.03	3.34	0.05
58	0.57	0.05	0.57	0.04	0.64	0.04	0.84	0.05
59	0.52	0.02	0.55	0.03	0.71	0.04	1.09	0.04
60	6.58	0.16	7.01	0.16	7.22	0.11	7.52	0.13
61	1.21	0.06	1.32	0.10	1.67	0.11	2.17	0.07
62	5.99	0.03	6.42	0.03	6.69	0.03	6.98	0.10
63	0.22	0.01	0.21	0.01	0.24	0.01	0.31	0.01
64	0.49	0.03	0.60	0.03	0.87	0.02	1.24	0.03
65	0.49	0.04	0.62	0.05	0.96	0.02	1.37	0.04
66	0.45	0.05	0.67	0.04	0.90	0.10	1.26	0.09
67	0.13	0.03	0.11	0.03	0.11	0.02	0.18	0.02
68	0.51	0.08	0.63	0.07	0.82	0.07	1.22	0.06
69	0.52	0.11	0.65	0.07	0.82	0.11	1.16	0.07
70	0.66	0.05	0.90	0.03	1.30	0.03	1.92	0.06
71	0.76	0.03	1.00	0.03	1.43	0.03	2.08	0.05
72	3.16	0.13	4.43	0.34	5.37	0.10	7.08	0.17
73	3.87	0.18	5.65	0.17	7.26	0.21	10.01	0.20
74	0.81	0.08	1.02	0.07	1.41	0.07	2.02	0.09
75	1.50	0.07	2.30	0.07	2.78	0.06	3.26	0.26
77	1.43	0.06	1.76	0.02	2.12	0.06	2.55	0.08
78	1.77	0.04	2.74	0.08	3.44	0.13	4.28	0.11
79	1.24	0.05	1.83	0.05	2.03	0.06	2.24	0.06
80	1.54	0.14	1.60	0.15	2.04	0.10	2.72	0.15
81	1.54	0.02	1.69	0.03	2.07	0.03	2.77	0.05
82	1.75	0.13	2.03	0.12	2.55	0.13	3.30	0.14
83	0.80	0.01	1.61	0.01	1.99	0.03	2.29	0.03
84	0.76	0.01	1.45	0.01	1.72	0.01	1.80	0.03
85	0.61	0.04	1.20	0.05	1.42	0.07	1.50	0.06

Table S3d. Related to Figure 4. Average relative D uptake at 1 minute, 10 minutes, 30 minutes and 120 minutes of exposure along with standard deviation values (SD) for apo MtATP-PRT at pH 9.

pH 9	D UPTAKE /Da							
peptide #	1 min	SD	10 min	SD	30 min	SD	120 min	SD
1	4.13	0.13	4.31	0.13	4.88	0.13	5.56	0.39
2	4.30	0.04	4.40	0.09	5.03	0.11	5.74	0.13
3	4.40	0.02	4.60	0.05	5.24	0.06	6.05	0.14
4	4.01	0.15	4.25	0.12	4.81	0.18	5.44	0.22
5	4.71	2.24	4.82	1.85	5.39	1.55	6.08	1.05
6	0.82	0.13	0.96	0.05	1.09	0.17	1.59	0.09
7	2.21	0.15	2.42	0.08	2.83	0.19	2.97	0.16
8	2.43	0.11	2.75	0.12	3.19	0.11	3.47	0.12
9	3.79	0.27	4.01	0.25	4.27	0.23	4.53	0.22
10	2.24	1.09	2.45	0.81	2.82	1.35	3.01	1.51
11	1.55	0.04	1.85	0.05	2.06	0.06	2.34	0.06
12	1.44	0.08	1.72	0.06	1.86	0.08	2.12	0.09
13	1.06	0.02	1.24	0.02	1.39	0.02	1.54	0.03
14	1.49	0.03	1.89	0.03	2.14	0.03	2.41	0.04
15	1.51	0.08	1.99	0.08	2.27	0.07	2.56	0.08
16	1.60	0.26	1.98	0.44	2.37	0.38	2.68	0.23
17	0.89	0.17	1.26	0.15	1.59	0.25	2.13	0.45
18	0.91	0.12	1.31	0.08	1.68	0.15	2.38	0.15
19	1.12	0.04	1.49	0.04	1.94	0.04	2.36	0.06
20	0.89	0.02	1.25	0.03	1.61	0.01	2.03	0.02
21	1.20	0.03	1.64	0.02	2.12	0.04	2.53	0.02
22	1.69	0.24	2.67	0.17	3.42	0.23	3.82	0.24
23	1.75	0.27	2.35	0.24	2.87	0.21	3.33	0.30
24	1.38	0.03	1.49	0.14	1.66	0.16	1.92	0.17
25	0.97	0.03	1.13	0.03	1.23	0.06	1.42	0.08
26	1.05	0.03	1.20	0.12	1.31	0.07	1.54	0.11
27	2.20	0.04	2.10	0.06	2.17	0.09	2.27	0.16
28	3.68	0.22	3.80	0.12	3.96	0.13	4.07	0.34
29	2.90	0.19	3.18	0.22	3.40	0.22	3.46	0.27
30	2.62	0.18	2.92	0.08	3.37	0.12	3.99	0.27
31	2.26	0.05	2.60	0.60	3.16	0.76	3.55	0.67
32	2.30	0.09	2.24	0.02	2.36	0.03	2.65	0.07
33	1.53	0.04	1.74	0.04	2.04	0.05	2.48	0.07
34	1.64	0.02	1.68	0.03	1.77	0.07	1.88	0.04
35	2.80	0.07	2.86	0.06	3.11	0.10	3.54	0.11
36	2.76	0.06	2.80	0.07	2.94	0.07	3.16	0.09
37	4.80	0.25	4.86	1.18	5.48	1.48	6.04	1.69
38	1.70	0.44	1.97	0.75	2.20	0.58	2.59	0.63
39	1.72	0.23	2.32	0.26	3.08	0.27	4.05	0.21
40	1.24	0.40	1.81	0.45	2.28	0.41	3.30	0.42
41	1.36	0.58	1.60	0.26	2.16	0.72	3.19	0.36
42	2.18	0.04	2.59	0.05	2.91	0.03	3.34	0.07
43	2.81	0.05	3.19	0.03	3.46	0.02	3.93	0.06
44	3.23	0.15	3.73	0.14	4.17	0.16	4.86	0.12
45	2.69	0.10	3.18	0.06	3.78	0.07	4.32	0.14

peptide #	1 min	SD	10 min	SD	30 min	SD	120 min	SD
46	1.60	0.09	1.72	0.06	1.89	0.06	2.17	0.07
47	2.45	0.03	2.82	0.02	3.32	0.02	3.70	0.06
48	1.90	0.18	2.26	0.13	2.68	0.13	3.47	0.16
49	2.00	0.51	2.55	0.50	3.01	0.46	3.69	0.49
50	2.31	0.14	2.65	0.13	3.17	0.22	3.95	0.15
51	1.12	0.06	1.38	0.03	1.78	0.11	2.31	0.05
52	0.62	0.11	0.83	0.06	1.61	0.36	1.71	0.08
53	1.10	0.13	1.09	0.10	1.52	0.08	2.11	0.10
54	6.82	0.07	7.00	0.11	7.72	0.10	8.69	0.19
55	5.00	0.03	5.21	0.07	5.67	0.07	6.22	0.15
56	5.05	0.03	5.22	0.05	5.47	0.07	5.83	0.14
57	3.27	0.03	3.48	0.04	3.62	0.04	3.82	0.10
58	0.67	0.04	0.77	0.05	0.94	0.04	1.23	0.06
59	0.73	0.10	0.91	0.06	1.19	0.06	1.66	0.09
60	7.13	0.13	7.29	0.08	7.51	0.13	7.98	0.16
61	1.33	0.07	1.66	0.11	1.94	0.13	2.32	0.06
62	6.65	0.05	6.70	0.07	6.84	0.10	7.15	0.15
63	0.97	0.05	1.31	0.05	2.10	0.08	3.02	0.17
64	1.84	0.07	2.56	0.08	3.85	0.07	4.84	0.10
65	2.09	0.59	2.85	0.50	4.56	0.31	5.62	0.34
66	2.19	0.47	3.07	0.52	4.68	0.27	5.81	0.26
67	1.13	0.19	1.34	0.21	1.67	0.19	2.28	0.07
68	1.40	0.11	1.99	0.19	3.24	0.08	4.19	0.06
69	1.87	0.39	2.63	0.48	4.22	0.33	5.55	0.32
70	1.09	0.03	1.61	0.04	2.41	0.04	2.64	0.07
71	1.57	0.73	2.39	0.71	3.25	0.60	3.86	0.81
72	5.51	0.14	6.81	0.11	8.42	0.27	8.53	0.37
73	8.21	0.23	10.36	0.22	12.37	0.18	13.18	0.38
74	1.01	0.11	1.49	0.12	2.21	0.11	2.48	0.13
75	3.72	0.46	4.17	0.48	4.68	0.60	4.82	0.70
76	5.58	0.05	6.71	0.04	7.77	0.09	8.31	0.17
77	1.77	0.11	2.12	0.08	2.53	0.13	2.86	0.11
78	4.32	0.16	4.95	0.20	5.22	0.26	5.52	0.08
79	1.81	0.63	2.19	0.59	2.23	0.47	2.48	0.56
80	1.74	0.08	2.41	0.20	4.29	0.14	5.67	0.20
81	1.76	0.04	2.18	0.06	3.60	0.11	4.57	0.12
82	1.94	0.13	2.53	0.18	4.03	0.19	5.10	0.32
83	1.11	0.06	2.13	0.08	3.75	0.09	4.67	0.11
84	1.02	0.05	1.94	0.07	3.31	0.12	4.29	0.09
85	0.88	0.06	1.65	0.10	2.85	0.14	3.65	0.12

Table S3e. Related to Figure 4. Average relative D uptake at 1 minute, 10 minutes, 30 minutes and 120 minutes of exposure along with standard deviation values (SD) for MtATP-PRT in presence of L-histidine at pH 9.

pH 9	D UPTAKE / Da							
peptide #	1 min	SD	10 min	SD	30 min	SD	120 min	SD
1	4.09	0.03	4.26	0.04	4.71	0.05	5.42	0.28
2	4.23	0.02	4.33	0.06	4.82	0.18	5.57	0.05
3	4.33	0.04	4.50	0.06	4.98	0.07	5.87	0.07
4	4.00	0.13	4.18	0.08	4.60	0.20	5.28	0.20
5	4.75	2.40	4.75	1.90	5.00	0.75	5.95	1.58
6	0.80	0.06	0.80	0.14	1.13	0.05	1.36	0.07
7	2.23	0.14	2.49	0.16	2.64	0.14	3.06	0.09
8	2.33	0.05	2.58	0.13	3.20	0.16	3.39	0.09
9	3.67	0.24	3.98	0.25	4.24	0.24	4.45	0.18
10	2.45	1.11	2.48	0.84	2.78	1.32	2.96	1.06
11	1.55	0.04	1.85	0.04	1.99	0.06	2.25	0.05
12	1.41	0.06	1.67	0.07	1.82	0.07	2.05	0.07
13	1.04	0.01	1.22	0.02	1.37	0.02	1.54	0.02
14	1.46	0.02	1.89	0.04	2.12	0.02	2.37	0.02
15	1.47	0.04	1.91	0.11	2.18	0.07	2.50	0.04
16	1.49	0.23	1.85	0.10	2.23	0.18	2.57	0.13
17	0.90	0.10	1.24	0.13	1.62	0.14	2.14	0.24
18	0.90	0.08	1.24	0.07	1.60	0.07	2.37	0.23
19	1.09	0.02	1.45	0.03	1.90	0.03	2.35	0.02
20	0.85	0.02	1.22	0.03	1.58	0.03	1.95	0.02
21	1.17	0.03	1.61	0.02	2.08	0.03	2.54	0.02
22	2.08	0.24	2.80	0.12	3.50	0.16	4.04	0.12
23	1.75	0.27	2.42	0.24	3.03	0.26	3.50	0.18
24	1.29	0.07	1.55	0.09	1.62	0.15	1.85	0.13
25	0.94	0.03	1.13	0.04	1.23	0.07	1.31	0.05
26	0.94	0.06	1.12	0.05	1.23	0.08	1.41	0.10
27	2.17	0.02	2.09	0.02	2.15	0.03	2.31	0.06
28	3.70	0.11	3.82	0.13	3.93	0.15	4.02	0.27
29	3.01	0.27	3.09	0.29	3.27	0.23	3.55	0.14
30	2.73	0.10	2.96	0.16	3.28	0.11	4.12	0.10
31	2.38	0.60	2.46	0.04	2.77	0.04	3.41	0.53
32	2.34	0.15	2.31	0.10	2.38	0.02	2.72	0.05
33	1.49	0.03	1.70	0.03	1.96	0.04	2.42	0.06
34	1.60	0.02	1.66	0.02	1.73	0.03	1.86	0.01
35	2.76	0.11	2.84	0.06	2.93	0.11	3.47	0.10
36	2.74	0.05	2.83	0.08	2.90	0.09	3.18	0.08
37	4.86	1.31	4.55	0.12	5.30	1.86	5.85	0.79
38	1.57	0.51	1.79	0.68	2.15	0.65	2.46	0.69
39	1.81	0.24	2.14	0.27	2.62	0.21	4.08	0.16
40	1.42	0.25	1.65	0.25	2.12	0.24	3.48	0.28
41	1.11	0.53	1.33	0.58	1.76	0.68	2.72	0.12
42	2.14	0.04	2.63	0.13	2.77	0.03	3.31	0.04
43	2.75	0.04	3.15	0.02	3.35	0.04	3.86	0.07
44	3.17	0.13	3.70	0.13	3.99	0.12	4.79	0.14
45	2.69	0.10	3.17	0.07	3.71	0.08	4.41	0.10

peptide #	1 min	SD	10 min	SD	30 min	SD	120 min	SD
46	1.58	0.09	1.67	0.07	1.87	0.09	2.15	0.05
47	2.43	0.01	2.83	0.02	3.29	0.06	3.70	0.02
48	1.88	0.12	2.19	0.13	2.43	0.15	3.39	0.11
49	1.96	0.68	2.29	0.67	2.80	0.71	3.46	0.63
50	2.34	0.19	2.62	0.18	2.99	0.22	3.94	0.19
51	1.09	0.06	1.33	0.03	1.58	0.10	2.23	0.15
52	0.71	0.04	0.76	0.04	0.97	0.09	1.65	0.05
53	0.96	0.10	1.12	0.05	1.26	0.08	2.00	0.14
54	6.77	0.05	6.99	0.07	7.46	0.07	8.58	0.17
55	4.83	0.10	5.22	0.06	5.57	0.06	6.16	0.07
56	5.03	0.02	5.22	0.04	5.45	0.04	5.82	0.07
57	3.23	0.05	3.46	0.03	3.56	0.05	3.76	0.04
58	0.71	0.03	0.81	0.11	0.88	0.19	1.28	0.09
59	0.69	0.08	0.79	0.06	1.03	0.08	1.62	0.08
60	7.20	0.15	7.35	0.08	7.52	0.11	7.97	0.14
61	1.40	0.05	1.72	0.12	1.91	0.11	2.44	0.04
62	6.65	0.03	6.75	0.05	6.93	0.04	7.15	0.03
63	0.90	0.06	1.12	0.06	1.70	0.06	2.90	0.08
64	1.69	0.17	2.20	0.04	3.30	0.07	4.80	0.05
65	1.85	0.65	2.43	0.53	3.79	0.57	5.42	0.35
66	2.04	0.51	2.64	0.55	4.15	0.34	5.75	0.17
67	1.16	0.26	1.24	0.24	1.53	0.20	2.28	0.11
68	1.28	0.06	1.70	0.08	2.77	0.04	4.15	0.08
69	1.75	0.37	2.41	0.42	3.66	0.41	5.26	0.14
70	1.03	0.04	1.46	0.08	2.19	0.02	2.66	0.06
71	1.51	0.68	2.06	0.81	2.85	0.71	3.61	0.67
72	5.13	0.09	6.48	0.18	7.99	0.19	8.79	0.31
73	7.38	0.22	9.82	0.23	11.84	0.21	13.24	0.16
74	0.98	0.12	1.42	0.12	2.07	0.13	2.50	0.12
75	3.59	0.47	3.97	0.49	4.58	0.49	4.60	0.56
76	5.00	0.09	6.42	0.04	7.40	0.03	8.29	0.05
77	1.73	0.08	2.13	0.06	2.52	0.10	2.91	0.09
78	3.86	0.07	4.73	0.09	5.07	0.19	5.40	0.26
79	1.56	0.70	2.03	0.64	2.08	0.65	2.46	0.75
80	1.64	0.17	2.06	0.18	3.39	0.17	5.56	0.23
81	1.71	0.03	2.03	0.02	2.97	0.08	4.53	0.08
82	1.80	0.18	2.25	0.14	3.37	0.16	5.10	0.31
83	1.05	0.03	1.95	0.03	3.14	0.02	4.78	0.03
84	0.98	0.03	1.78	0.01	2.82	0.02	4.32	0.05
85	0.83	0.06	1.47	0.11	2.36	0.10	3.66	0.15
