# Supplementary material for: Hybrid Mass Spectrometry Approaches to Determine How L-Histidine Feedback Regulates the Enzyzme MtATP-Phosphoribosyltransferase
Source: Structure. 2017 May 2;25(5):730–738.e4. doi: 10.1016/j.str.2017.03.005 (PMC5415358; doi:10.1016/j.str.2017.03.005)
Supplement: Document S2. Article plus Supplemental Information [file mmc2.pdf]

Structure

Article

# Hybrid Mass Spectrometry Approaches to Determine How L-Histidine Feedback Regulates the Enzyzme MtATP-Phosphoribosyltransferase

## Graphical Abstract

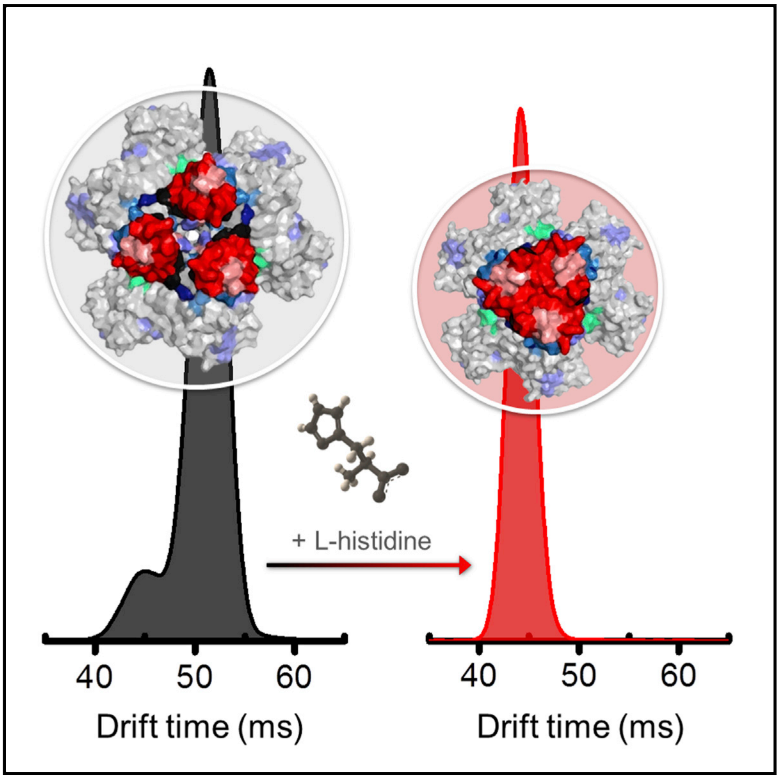


## Highlights

- Hybrid MS approaches map global and local conformational changes in MtATP- PRT
- IM-MS shows hexameric MtATP-PRT to undergo conformational change on L-histidine binding
- HDX-MS maps conformational changes to regions close to and remote from the active site

## Authors

Kamila J. Pacholarz, Rebecca J. Burnley, Thomas A. Jowitt, ..., Richard J. Taylor, Luiz Pedro Sório de Carvalho, Perdita E. Barran

## Correspondence

luiz.carvalho@crick.ac.uk (L.P.S.d.C.), perdita.barran@manchester.ac.uk (P.E.B.)

## In Brief

MtATP-phosphoribosyltransferase (MtATP-PRT) catalyzes the first step in *Mycobacterium tuberculosis* L-histidine biosynthesis. Pacholarz et al. use mass spectrometry approaches to show that MtATP-PRT is a hexamer, and use measurements at different pH values to demonstrate that L-histidine allosteric effect does not alter the oligomeric state of the enzyme.

Pacholarz et al., 2017, Structure *25*, 730–738
May 2, 2017 © 2017 The Author(s). Published by Elsevier Ltd.
http://dx.doi.org/10.1016/j.str.2017.03.005

Structure
Article

# Hybrid Mass Spectrometry Approaches to Determine How L-Histidine Feedback Regulates the Enzyme MtATP-Phosphoribosyltransferase

Kamila J. Pacholarz,[1,2] Rebecca J. Burnley,[3] Thomas A. Jowitt,[4] Victoria Ordsmith,[2] João Pedro Pisco,[5] Massimiliano Porrini,[2] Gérald Larrouy-Maumus,[5,6] Rachel A. Garlish,[3] Richard J. Taylor,[3] Luiz Pedro Sório de Carvalho,[5,*] and Perdita E. Barran[1,7,*]
[1]Michael Barber Centre for Collaborative Mass Spectrometry, Manchester Institute of Biotechnology, School of Chemistry, 131 Princess Street, Manchester M1 7DN, UK
[2]School of Chemistry, University of Edinburgh, David Brewster Road, Edinburgh EH9 3FJ, UK
[3]UCB Celltech, 216 Bath Road, Slough SL1 3WE, UK
[4]The Biomolecular Analysis Facility, Faculty of Life Sciences, University of Manchester, Oxford Road, Manchester M13 9PT, UK
[5]Mycobacterial Metabolism and Antibiotic Research Laboratory, The Francis Crick Institute, 1 Midland Road, London NW1 1AT, UK
[6]Present address: Centre for Molecular Bacteriology and Infection, Imperial College, London SW7 2AZ, UK
[7]Lead Contact
*Correspondence: luiz.carvalho@crick.ac.uk (L.P.S.d.C.), perdita.barran@manchester.ac.uk (P.E.B.)
http://dx.doi.org/10.1016/j.str.2017.03.005

## SUMMARY

**MtATP-phosphoribosyltransferase (MtATP-PRT) is an enzyme catalyzing the first step of the biosynthesis of L-histidine in *Mycobacterium tuberculosis*, and proposed to be regulated via an allosteric mechanism. Native mass spectrometry (MS) reveals MtATP-PRT to exist as a hexamer. Conformational changes induced by L-histidine binding and the influence of buffer pH are determined with ion mobility MS, hydrogen deuterium exchange (HDX) MS, and analytical ultracentrifugation. The experimental collision cross-section ($^{DT}CCS_{He}$) decreases from 76.6 to 73.5 nm$^2$ upon ligand binding at pH 6.8, which correlates to the decrease in CCS calculated from crystal structures. No such changes in conformation were found at pH 9.0. Further detail on the regions that exhibit conformational change on L-histidine binding is obtained with HDX-MS experiments. On incubation with L-histidine, rapid changes are observed within domain III, and around the active site at longer times, indicating an allosteric effect.**

## INTRODUCTION

Naturally occurring allosteric sites in metabolic enzymes controlled through feedback effectors are one of nature's ways of regulating biochemical pathways. Physiological inhibitors may serve as templates for chemically diverse, small-molecule allosteric inhibitors sought after as regulators for synthetic biology, as therapeutics for diseases, or as probes in both chemical genetics and chemical biology. In addition, allosteric sites become attractive drug targets when the active sites of an enzyme are "not targetable" or have poor "ligandability" (Edfeldt et al., 2011; Hardy and Wells, 2004; Surade and Blundell, 2012).

A remarkable example of an allosterically regulated enzyme is ATP-phosphoribosyltransferase (ATP-PRT). MtATP-PRT catalyzes the first step in the biosynthesis of L-histidine in *Mycobacterium tuberculosis* (*Mt*). Being a feedback allosterically regulated enzyme, it is inhibited by the end product of the pathway, i.e., L-histidine (Pedreño et al., 2012).

Two mechanisms have been proposed to describe the allosteric effects induced by ligands on multimeric proteins. In the first, binding of the ligand causes a change in conformation, which then stabilizes one oligomeric state, causing for example a shift from a dimeric to a hexameric form (Figures 1A–1C). In the second, no changes in the oligomer order are observed but more localized structural changes in the complex do take place, e.g., loop and domain motions, which may tighten the overall structure (Figure 1B). Experimental discrimination between these two cases is not always straightforward. For MtATP-PRT, Cho et al. (2003) determined crystal structures of the apo form at pH 6.5 and in complex with L-histidine and AMP at pH 5.6. The authors suggested that MtATP-PRT is an active dimer that assembles into an inactive hexamer upon L-histidine binding (Figure 1D) (Cho et al., 2003). Moreover, it was found that L-histidine binds ~30 Å from the active site inducing a significant conformational change and supporting an allosteric inhibition mechanism. Cartoon representations of MtATP-PRT from this crystal structure projected along the 3-fold axis are shown in Figure 1A and the 2-fold axis where the C terminus is shown in the front plane in Figure 1B (Cho et al., 2003). The major conformational change in the L-histidine bound form is a significant twist of domain III, where the allosteric site is located, with respect to domains I and II causing steric hindrance in the active site. L-Histidine molecules were reported to bind to domain III clusters at both ends of each dimer, with a stoichiometry of 1:2 dimer:L-histidine to stabilize the hexameric form. Binding of L-histidine reorients some of the key residues in the active site.

An alternative inhibition mechanism to the dimer to inactive hexamer (Figure 1E) was proposed by Pedreño et al. (2012) and is supported by size-exclusion chromatography (SEC). In this, MtATP-PRT is thought to be present mostly in the

 © 2017 The Author(s). Published by Elsevier Ltd.
This is an open access article under the CC BY license (http://creativecommons.org/licenses/by/4.0/).

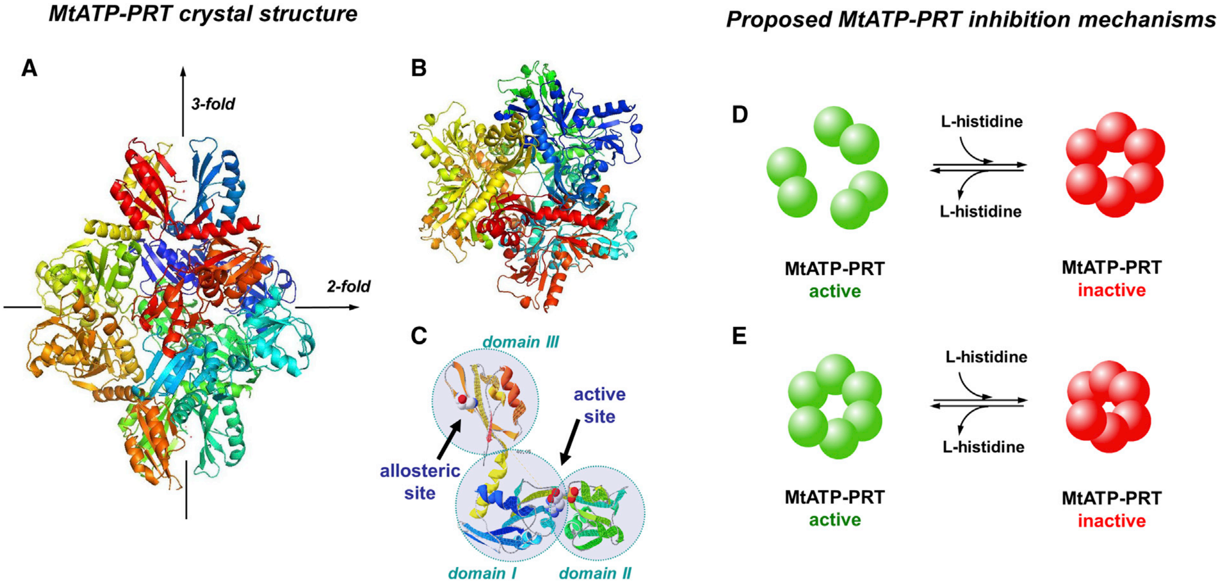


**Figure 1. Structure and Proposed Inhibition Mechanisms of MtATP-PRT**

(A and B) Graphical representation of the hexameric apo MtATP-PRT X-ray crystal structure along (A) the 3-fold symmetry axis and (B) the 2-fold symmetry axis. Data available in the PDB consist of MtATP-PRT dimer (PDB: 1NH7); here the structure has been assembled into a hexamer using PISA (Krissinel and Henrick, 2007; Krissinel, 2010).

(C) The MtATP-PRT monomer consists of three contiguous domains indicated with teal circles: I, II, and III (PDB: 1NH8). Location of the allosteric site (with L-histidine) and the active site (with AMP) are indicated with black arrows. A crystallographic study by Cho et al. (2003) presents monomeric MtATP-PRT as an elongated molecule composed of 10 α helices and 15 β strands situated across three continuous domains (C). Domain I (residues 1–90, 175–184, and 194–211) consists of a central β sheet (four parallel β strands, β1, β3–5; and two anti-parallel strands, β2 and β11) surrounded by three α helices (α1–3). Domain II (residues 91–174) has four parallel β strands (β7–10), one anti-parallel β strand (β6), and two α helices (α4 and 5). Domain III (residues 212–284) is composed of one β sheet (four anti-parallel β stands, β12–15) and two α helices (α9 and 10). The catalytic core of MtATP-PRT is located in a highly negatively charged cavity between domains I and II. L-histidine is held at the allosteric site by interactions with the carboxyl group of aspartic acid at position 218 (Asp218), the hydroxyl group of threonine at position 238 (Thr238), and the backbone amide oxygen from alanine at position 273 (Ala273) in domain III (Cho et al., 2003).

(D and E) Proposed mechanisms of MtATP-PRT allosteric inhibition with L-histidine postulated by Cho et al. (2003) (D) and Pedreño et al. (2012) (E).

See also Figure S1.

hexameric form in solution, both in the presence and absence of the L-histidine or ATP. Similarly, no shift in the oligomeric state was observed for the hexameric homologous enzyme from *Salmonella enterica* (Martin, 1963; Voll et al., 1967). These findings are in contrast with results reported by Cho et al. (2003). Pedreño et al. (2012) speculated that the observed differences are possibly due to different experimental conditions, and that lower pH values could lead to dissociation of MtATP-PRT into dimers as there is no evidence that the dimer represents a kinetically competent form of MtATP-PRT (the activity drops below pH 8; see Supplemental Information, Figure S1). A proposed mechanism (Figure 1E) envisions MtATP-PRT as a functional hexamer where conformational changes have to be invoked to explain the allosteric inhibition. To unambiguously and independently define the mechanism of inhibition, i.e., subtle conformational changes versus changes in oligomerization, experimental methods that probe native conformations along with conformational changes are desirable.

Native mass spectrometry (MS) is a fast and sensitive methodology for analysis of intact proteins and non-covalent protein complexes (Konijnenberg et al., 2013; Marcoux and Robinson, 2013; Pacholarz et al., 2012; Pukala et al., 2009) and has recently been applied in the field of allosteric enzyme regulation to distinguish between the classic models of cooperativity (Beveridge et al., 2016; Dyachenko et al., 2013). By inclusion of the related technique of ion mobility with mass spectrometry (IM-MS), it is possible to obtain the collision cross-sections (CCS) of proteins in addition to the mass to charge (*m/z*) information, which provides insight into conformational changes occurring, for example, upon ligand binding.

Experimental CCSs are often benchmarked to theoretical CCSs derived from structures based on nuclear magnetic resonance or X-ray crystallography, which facilitates analysis of the different conformational populations accessible to the protein complex under investigation (Jurneczko and Barran, 2011). Hydrogen deuterium exchange (HDX) coupled with MS can be usefully applied to examine conformational changes in solution. Highly dynamic and disordered regions or those with increased solvent accessibility undergo rapid deuteration resulting in a large mass increase (Iacob et al., 2011; Skinner et al., 2012; Zhang et al., 2010). Both MS techniques enable structural studies on significantly shorter timescales than with conventional biophysical approaches and require less material; as an example, we have used approximately 4 mg of MtATP-PRT to carry out the MS experiments reported here.

The collected data showcase how MS methodologies can reveal MtATP-PRT as a functional hexamer and delineate where global and local conformational changes are invoked

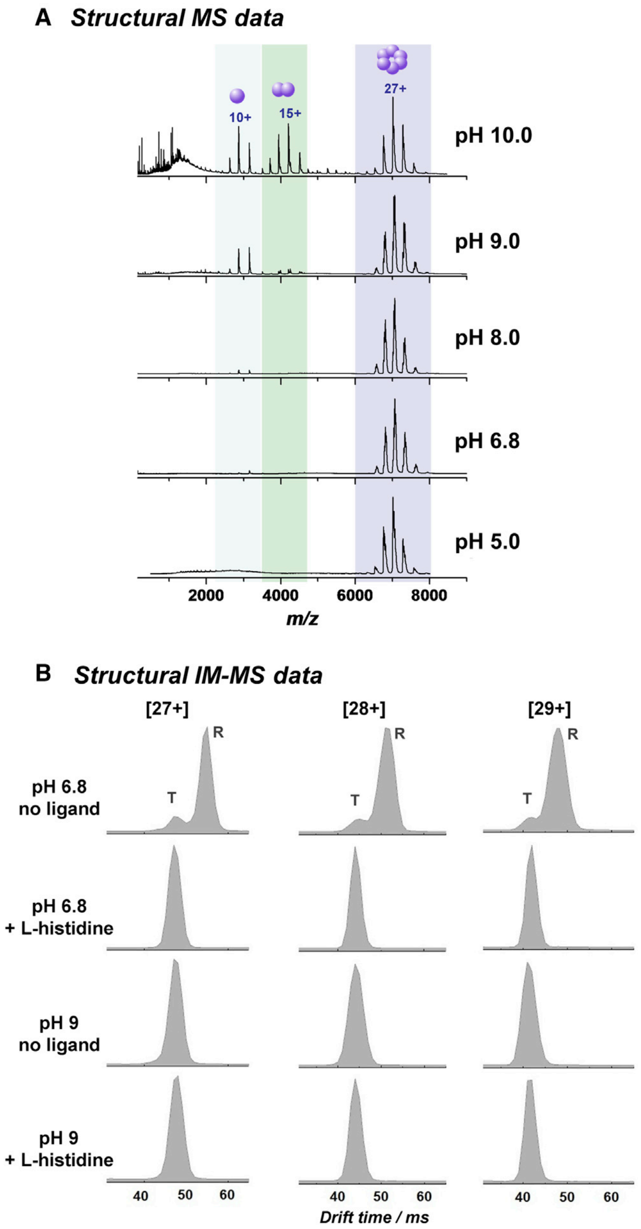


**Figure 2. MS and IM-MS Data Supporting Global Conformational Changes in MtATP-PRT**

(A) nESI mass spectra of 20 μM MtATP-PRT (hexamer concentration) in 100 mM ammonium acetate at pH 5.0, pH 6.8, pH 8.0, pH 9.0, and pH 10.0, showing the effect of pH on the oligomeric state of the enzyme.

(B) Drift-time distributions of [27+], [28+], and [29+] charge states of MtATP-PRT in the presence and absence of L-histidine in 100 mM ammonium acetate at pH 6.8 and at pH 9.0.

Also see Figures S2 and S4 and Table S1.

for allosteric inhibition. Native IM-MS indicates a considerable pH-dependent conformational change in the hexamer, which given the relative mass of the protein (189 kDa) versus L-histidine (155 Da) allows the action of binding to be determined more convincingly than with native mass analysis alone. We go on to describe a novel titration method to determine the Hill number to assess the cooperativity of binding. This indicates that the binding of the first four L-histidine molecules is different from the next two, and we can infer that the conformational change occurs between 4 and 6 equivalents, and that the binding is cooperative. This MS method has never been used before.

The gas-phase work is supported by solution assays using HDX-MS, which shows the regulation to be allosteric, and by analytical ultracentrifugation (AUC), which proves less informative than IM-MS analysis. Intriguingly, the conformational change at the active site occurs much later (120 min) than the change close to the inhibitor binding region, and this highlights the benefit of using HDX-MS to measure allostery. Both the IM data and the AUC data are supported by modeling, which in turn provides a scale for the magnitude of the conformational change.

The stand out result is that we can measure the action of binding of a 155 Da natural inhibitor to the hexameric MtATP-PRT protein of ~190 kDa because of the conformational change that it induces. We showcase the advantage of MS-based analytical approaches to measure the conformational rearrangements that occur to this protein as L-histidine binds, and contrast these with the more conventional methodology of SEC.

## RESULTS AND DISCUSSION

### MtATP-PRT Exists Mainly in a Hexameric Form under Physiological Conditions

The oligomeric state of apo MtATP-PRT as a function of pH was investigated using nano-electrospray ionization (nESI)-MS (Figure 2A). Signals corresponding to the hexameric form of MtATP-PRT were observed between ~*m/z* 6,500–7,500, as a charge-state distribution (CSD) from [25+] to [29+] centered on [27+]. From this, the experimentally determined mass of the MtATP-PRT hexamer was found to be 189,374 ± 23 Da, slightly higher than the theoretical mass (189,090 Da), which can be attributed to residual solvent and salt adducts. At pH 6.8, a minor amount of monomeric MtATP-PRT is also present (the CSD around *m/z* 3,000). The experimentally determined mass of the MtATP-PRT monomer of 31,516 ± 11 Da matches closely the theoretical average mass determined for MtATP-PRT monomer (31,515.6 Da), indicating that, unlike the hexamer, this monomer does not possess a fold that retains non-covalent adducts (upon application of harsh source conditions). The intensity of the monomeric signal increases as the pH is raised. At pH 9.0, dimeric MtATP-PRT is also detectable (~*m/z* 3,900–4,600, centered at [15+] charge state), which also becomes more prominent with increasing pH. These experiments support the hypothesis that MtATP-PRT is present in solution predominantly in a hexameric form in the absence of any ligands, in excellent agreement with Pedreño et al. (2012).

### Insights into Ligand Binding Stoichiometry from Native MS Experiments: Stoichiometry of L-Histidine Binding

X-ray crystallography experiments indicate up to six L-histidine molecules can bind to one MtATP-PRT hexamer (Cho et al., 2003). However, L-histidine occupancy (partial or full) was not well described, and we chose to investigate this with an MS

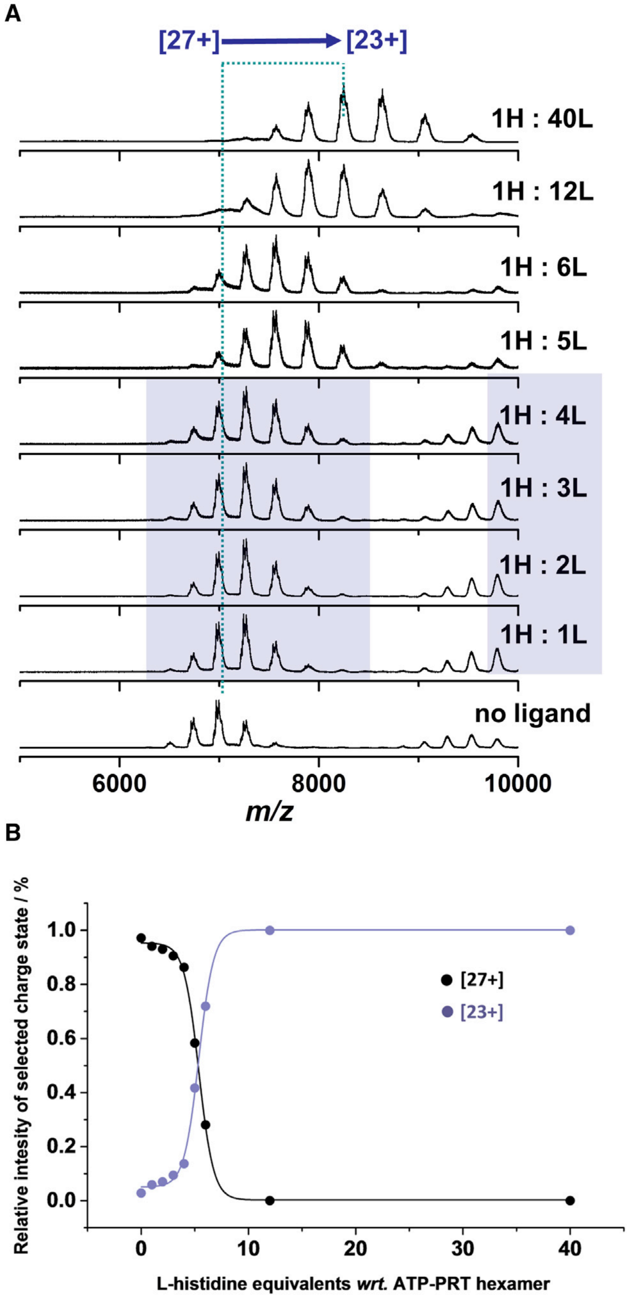


**Figure 3. L-Histidine Titration into MtATP-PRT**

(A) Titration of L-histidine into MtATP-PRT at 1–40 L-histidine equivalents to 1 MtATP-PRT hexamer in 100 mM ammonium acetate (pH 6.8) acquired at a constant cone voltage (high CV). A greater shift in the CSD profiles is observed along with increasing concentration of L-histidine.

(B) Plot of the decrease in intensity of the [27+] charge state and the increase in intensity of the [23+] charge state as a function of increasing amounts of L-histidine.

Also see Figure S3.

titration assay. Twelve equivalents of L-histidine were added to 1 equivalent of MtATP-PRT hexamer in 100 mM ammonium acetate (pH 6.8) and incubated for 1 hr at room temperature (Figures 3 and S3). The mass of the MtATP-PRT hexamer (189 kDa) is far larger than the mass of a single L-histidine (155.15 Da), and this large discrepancy precludes resolution of bound from unbound species. Despite this, upon addition of L-histidine and under gentle source conditions (low cone voltage [CV]; see Supplemental Information), a shift in the peak center of the protein complex peak is apparent. The mass spectra show a mass increase of ~1.2 kDa, corresponding to the binding of ~8 molecules, implying super-stoichiometric and perhaps non-specific binding (Figure S3A). Nevertheless, under these conditions, a significant amount of residual solvent is still present, precluding a precise assessment of binding stoichiometry. Even under harsher desolvation conditions (high CV) (Figure S3A), a significant amount of solvent is present, which again could bias correct stoichiometry determination. In the presence of the ligand at high CV (Figure S3B), a significant shift in the charge-state envelope was observed (from [27+] to [24+]) which does not occur with the apo hexamer. This CSD shift is found to be pH dependent (Figure S2), and we postulate that under harsher conditions, the ligand dissociates from the complex in source and leaves as a protonated molecule, which results in a reduction in the net charge of the protein hexamer. No increase in the monomer signal was observed at high CV, indicating that ligand binding is more readily disrupted than the protein:protein interfaces.

The CSD shift arising from in-source ligand loss gives insight into ligand binding affinities (Figure 3A). No differences in mass between apo MtATP-PRT and those incubated with L-histidine were noted, implying that all L-histidine molecules have dissociated under these harsh source conditions. With 1–4 L-histidine equivalents, a minor change in the CSD (from centered on [27+] to [26+]) of one charge is noted, and the lower four mass spectra (Figure 3C in purple box) display the same charge-state profiles, implying that each of these first four ligands binds in an equivalent way. As the amount of L-histidine is increased to 5 and 6 equivalents, the CSD shift is now two in both cases (from [27+] apo to [25+]), suggesting that the binding affinity and/or the protein conformation has now altered. When more L-histidine is titrated in, to 12 and 40 equivalents, only very small further shifts in the CSD are observed to a maximum of four indicating that after 6 equivalents, the ligand no longer binds specifically.

The Hill number ($n^H$) provides a way to quantify the degree of cooperativity and number of ligand binding sites (Coval, 1970; Heck, 1971). L-Histidine has been shown to inhibit MtATP-PRT with an $n^H$ of 1.5 (Pedreño et al., 2012). Using our CSD shift data, we are able to construct a plot (Figure 3B) that provides good evidence for cooperatively; here we have taken the [23+] charge state as representative of a fully L-histidine loaded form and [27+] to represent the apo form. This is supported by the data in Figure 3A. Using this analysis, we observe saturation of binding at ~5.3 ligand equivalents, in good agreement with the findings of others. When partial occupancy is detected, different binding affinities can also be observed. These experiments suggest that the first four L-histidine molecules have different affinities from the final two (Figure 3).

## The Effect of pH on the Oligomeric State of MtATP-PRT in the Presence of L-Histidine

The pH of the buffer solution affects the oligomeric state of MtATP-PRT (Figure 2A). Incubation for 240 min with L-histidine

(298K) reduces the amount of observed monomeric and dimeric species (Figures S2C and S2D). At pH 9.0, a significant amount of MtATP-PRT monomer and a marginal amount of dimer are present (bottom spectrum in Figure S2C). After 30 min of incubation with 12 equivalents of L-histidine, the intensity of both lower-order species decreases and is no longer detectable after a prolonged incubation period of 240 min.

At pH 10.0, an even greater proportion of monomeric and dimeric MtATP-PRT was observed (Figure S2D). Here, a 30 min incubation with L-histidine does not reduce the presence of either species. A longer incubation period is required to achieve a similar effect, and a decrease of these low-order oligomeric forms is only observed after 240 min. The high pH data are enacting the mechanism of Cho et al. (2003), that MtATP-PRT is an active dimer that assembles into an inactive hexamer upon L-histidine binding, but Cho et al. conducted their experiments at pH 6.5 (apo) and pH 5.6 (with L-histidine and AMP), conditions under which MtATP-PRT is kinetically inactive (see Figure S1) (Pedreño et al., 2012), and where we see scant evidence for dimer. L-Histidine does have a stabilizing effect on MtATP-PRT at high pH, regardless of whether it only binds to and does not inhibit the enzyme.

### Probing Conformational Changes Induced by Ligand Binding and Environmental Changes with IM-MS

Cho et al. (2003) reported on a large shift of domain III of MtATP-PRT with respect to domains I and II when comparing the dimeric form with hexameric MtATP-PRT formed in the presence of L-histidine and AMP. Although the dimeric form, described by Cho et al. is not observed here near neutral pH, the domain shift could still take place, generating an alternative hexamer, which in turn could be inhibited. Gel filtration studies by Pedreño et al. (2012) indicated MtATP-PRT to be present mostly in the hexameric form, and there was some evidence for subtle conformational changes upon L-histidine addition. Here, IM-MS, both drift-tube (DT) IM-MS and traveling wave IM (TWIMS) MS, are employed to more conclusively probe native conformations and conformational changes of MtATP-PRT.

### Determination of $^{DT}CCS_{He}$: Linear DT-IM-MS

Arrival time distributions (ATD) were recorded following mobility separation at 300 K and converted into collision cross-section distributions ($^{DT}CCSD_{He}$) as described in the Supplemental Information. In the presence of L-histidine, MtATP-PRT $^{DT}CCS_{He}$ is more compact, and this conformational tightening may be attributed to the shift of domain III. Changes in the mean $^{DT}CCS_{He}$ from 75.2 $nm^2$ to 73.6 $nm^2$, from 76.6 $nm^2$ to 73.5 $nm^2$, and from 76.4 $nm^2$ to 72.4 $nm^2$ were determined upon L-histidine binding for [27+], [28+], and [29+] charge states, respectively. These results show tightening of the hexamer upon inhibition by L-histidine.

### Tracing Ligand Binding and pH-Dependent Conformations

Conformational changes of MtATP-PRT due to ligand binding and pH were also explored with TWIMS-MS, which has higher mobility resolution than our DT-IM-MS instrument. ATDs for [27+], [28+], and [29+] charge states of apo MtATP-PRT and MtATP-PRT with L-histidine at pH 6.8 and at pH 9.0 are shown in Figure 2B. Apo MtATP-PRT at pH 6.8 presents clearly in two conformers, which we assign as tense (T) and relaxed (R). The relaxed conformation is significantly higher in abundance compared with the tense conformation, which constitutes about 12% of the total ion population for each charge state. Upon addition of 12 equivalents of L-histidine, R MtATP-PRT is no longer observed. The total ion population shifts to shortened drift times and matches the drift-time profile of T (for apo MtATP-PRT) at pH 6.8. This conformational tightening upon ligand binding is in agreement with the absolute $^{DT}CCS$ values reported above with DT-IM-MS and previous work with gel filtration and X-ray crystallography studies (Cho et al., 2003; Pedreño et al., 2012). Interestingly, under the same instrumental conditions, apo MtATP-PRT at pH 9.0 appears to be already in the T form, similar to the L-histidine-bound MtATP-PRT at pH 6.8. Upon addition of L-histidine, the drift time remains constant, suggesting no further changes in conformation take place. This trend is observed across all charge states. These results indicate a potential disconnect between conformational tightening and inhibition, induced by L-histidine at lower and higher pH. Future work will vary the pH over smaller steps and monitor the conformational change to more precisely locate the critical pH for the T to R transition.

### Conformational Changes Probed with Solution-Phase Technique: Analytical Ultracentrifugation

Sedimentation coefficient distributions of MtATP-PRT acquired at pH 6.8 and pH 9.0 are shown in Figure S4. The sedimentation coefficient values ($s_{20,w}$) of MtATP-PRT at pH 6.8 ± L-histidine were determined to be 8.58 ± 0.14 S and 8.22 ± 0.26 S, respectively (Figure S4A), along with a small amount of aggregate observed between 10 and 12 S. This increase in $s_{20,w}$ upon binding of L-histidine is indicative of conformational change and the adoption of a more compact structure. A narrowing of the $s_{20,w}$ distribution is observed upon L-histidine addition, suggesting that its presence may be causing MtATP-PRT to be more structurally constrained. The sedimentation coefficient values of MtATP-PRT at pH 9.0 in the presence and absence of L-histidine were determined to be 8.09 ± 0.18 S and 8.15 ± 0.14 S, respectively (Figure S4B). Here, the difference in $s_{20,w}$ between ligand-free and ligand-bound MtATP-PRT is significantly smaller, suggesting close structural resemblance of the two species, in agreement with the IM-MS and HDX-MS results. Minor differences in $s_{20,w}$ of apo MtATP-PRT at pH 6.8 and pH 9.0 are most likely due to an effect of the buffer pH on the degree of bound water rather than conformational changes. It is worth noting that the small population of lower-order species at $s_{20,w}$ = 6.41 S observed at pH 9.0 is no longer detected after the addition of L-histidine. Native MS experiments showed monomeric and dimeric MtATP-PRT present at pH 9.0 to similarly decrease in intensity after incubation with L-histidine (Figure S2C). In summary, AUC data are in agreement with results obtained from IM-MS and HDX-MS studies, i.e., detection of subtle conformational tightening of MtATP-PRT at pH 6.8 upon L-histidine addition noted, however, no evident conformational changes on L-histidine binding are found at pH 9.0.

### Conformational Differences Derived from the X-Ray Crystal Structures and a Bead Model: Theoretical $CCS_{He}$

The conformational change of MtATP-PRT is also found from $CCS_{He}$ calculated from the available X-ray crystal structures

(Shvartsburg et al., 1998). The theoretical $CCS_{He}$ of hexameric apo MtATP-PRT and Mt-ATP-PRT:L-histidine:AMP complex were found to be 94.0 $nm^2$ and 90.7 $nm^2$, respectively. The experimentally determined $^{DT}CCS_{He}$ are significantly (~23%) smaller than the theoretical $CCS_{He}$. We anticipate such compaction on desolvation as previously reported (Pacholarz et al., 2014), but notably this correlates with the finding that the theoretical $CCS_{He}$ of apo MtATP-PRT is also larger than the $CCS_{He}$ of inhibited MtATP-PRT. The difference between the gas-phase minimized crystal structures is 3.3 $nm^2$, which agrees well with the change in experimental $^{DT}CCS_{He}$ of 2.9 $nm^2$.

We have also performed bead modeling on both apo and holo crystal structure (see Supplemental Information, Table S1) and also found a small decrease in size upon L-histidine binding. The values found support the sedimentation results as well as our IM-MS data. A greater difference is found from the experimental AUC data than from the crystallographic input structures. The $R_g$ values from the bead models are found to be exactly the same (3.75 nm), whereas the sedimentation coefficient differs by 0.16 S (cf. 0.38 S found experimentally). This implies that there is a more significant difference in the hydration of the hexamer between the holo and apo forms than in the crystal structures. Akin to findings from ion mobility, the calculated diffusion coefficient of the apo form is smaller than that for the holo form (4.50 versus 4.54 cm $s^{-2}$) also showing the holo form to be smaller. The total surface area of the hexamers found from the bead models differ by 2.2%, again on the same order as $^{DT}CCS_{He}$ differences found experimentally.

## Mapping Conformational Changes on Ligand Binding with HDX-MS

HDX experiments were carried out on MtATP-PRT in the presence and absence of L-histidine (12 ligand equivalents to 1 hexamer) at pH 6.8 and 9.0. Percentage differences in D exchange between apo and L-histidine bound MtATP-PRT at pH 6.8 were calculated and applied to the available X-ray crystal structures PDB: 1NH7 (apo MtATP-PRT) and PDB: 1NH8 (MtATP-PRT + AMP + L-histidine) as shown in Figure 4A. Residues 226–259 located in the region of significant changes within the first minute of HDX are highlighted in red and encompass residues identified to be involved in direct binding with L-histidine: D228, L244, S246, T248, L263, and A283 (black). Residues for which differences in HDX were found on a longer timescale (120 min) are highlighted in three shades of blue of increasing intensity corresponding to greater changes. The greatest changes (difference of 20% and higher) in HDX between L-histidine-free and L-histidine-bound MtATP-PRT evident on a longer timescale were located near the allosteric site (210–223 and 275–285). Slightly less extensive but still notable (10%–20%) changes were found in regions surrounding the active site positioned in the cavity between domains I and II (44–55, 117–129, 152–164); which in Figure 4A are marked in teal green for residues involved in direct binding of AMP and lime green for residues proposed to be involved in ATP and PRPP binding.

Cho et al. (2003) reported a major conformational change upon L-histidine binding originating from a significant twist of domain III with respect to domains I and II, causing steric hindrance in the active site. HDX data support this hypothesis; we see over 30% change in uptake within the $\alpha$ helix between domains I and III (212–223). This implies conformational change induced by allosteric inhibitor binding, resulting in limited access to the active site and/or reorienting residues responsible for interactions with both of the substrates: ATP and PRPP. Table S2 lists residues and peptides undergoing notable changes in D exchange between ligand-free and ligand-bound MtATP-PRT states, as well as residues involved in interactions with inhibitors and substrates determined by Cho et al. (2003).

Illustrative D uptake curves of four chosen peptides of MtATP-PRT (green) and MtATP-PRT:L-histidine complex (red) are shown in Figure S5. The amount of D uptake varies along the MtATP-PRT sequence: regions spanning 44–51, 165–179, and 242–259, all of which are located on the surface of domains I, II, and III, respectively, experience higher deuteration on a short timescale (1 min) in comparison with residues 120–129, which are somewhat buried within domain II. Upon addition of L-histidine at pH 6.8, the amount of D uptake is reduced in the region within domain III (residues 242–259; Figure S5, bottom plot) assigned as the allosteric site of L-histidine binding. As L-histidine binds, this region becomes protected from the deuterated solvent, which significantly slows down the HDX reaction. Although the absolute D exchange increases with the HDX reaction time, the relative difference between the two species remains constant, suggesting it is protection of a particular residue/s that is responsible for the observed decrease.

Changes in HDX between ligand-free and ligand-bound MtATP-PRT are also observed on longer timescales (120 min of HDX). Residues 120–129, located near the AMP binding site and proposed ATP and PRPP binding site, show no significant change in D exchange at 1 min of HDX. At longer HDX times, however, a decrease in D exchange is noted for the L-histidine bound form. Such differences likely originate from conformational changes induced by ligand binding, which restrict protein dynamics and limit deuteration at extended exposure times rather than from direct interaction with the binding molecule.

By contrast, no differences in D exchange were found between apo- and L-histidine-bound MtATP-PRT at pH 9.0, neither around the allosteric region due to L-histidine binding nor near the active site. This indicates that conformational changes observed at pH 6.8 do not occur at pH 9.0, in agreement with the IM-MS data, and/or that, at this pH, L-histidine does not bind to MtATP-PRT. Average D exchange values, along with SD values (based on three experimental replicas) across four samples and four time points for each peptide identified are provided in the Supplemental Information.

The difference in absolute D uptake between apo MtATP-PRT and MtATP-PRT in the presence of L-histidine at four exposure time points is plotted in Figure S6 for each peptide along the x-axis from the N to C terminus, at pH 6.8 and pH 9.0. This shows how changes occurring on a fast timescale (observable <1 min) associated with direct L-histidine binding take place within domain III (peptides 70–85) at pH 6.8 (Figure S6A); whereas the other changes occur on longer timescales (most obvious at 120 min), suggesting an induced conformational change near the active site (peptides 35–60, 80–85) proceeding at a slower rate. It has been suggested that HDX changes at different time points as a way of differentiating between small-molecule binding and protein structural events (Krishnamurthy et al., 2013;

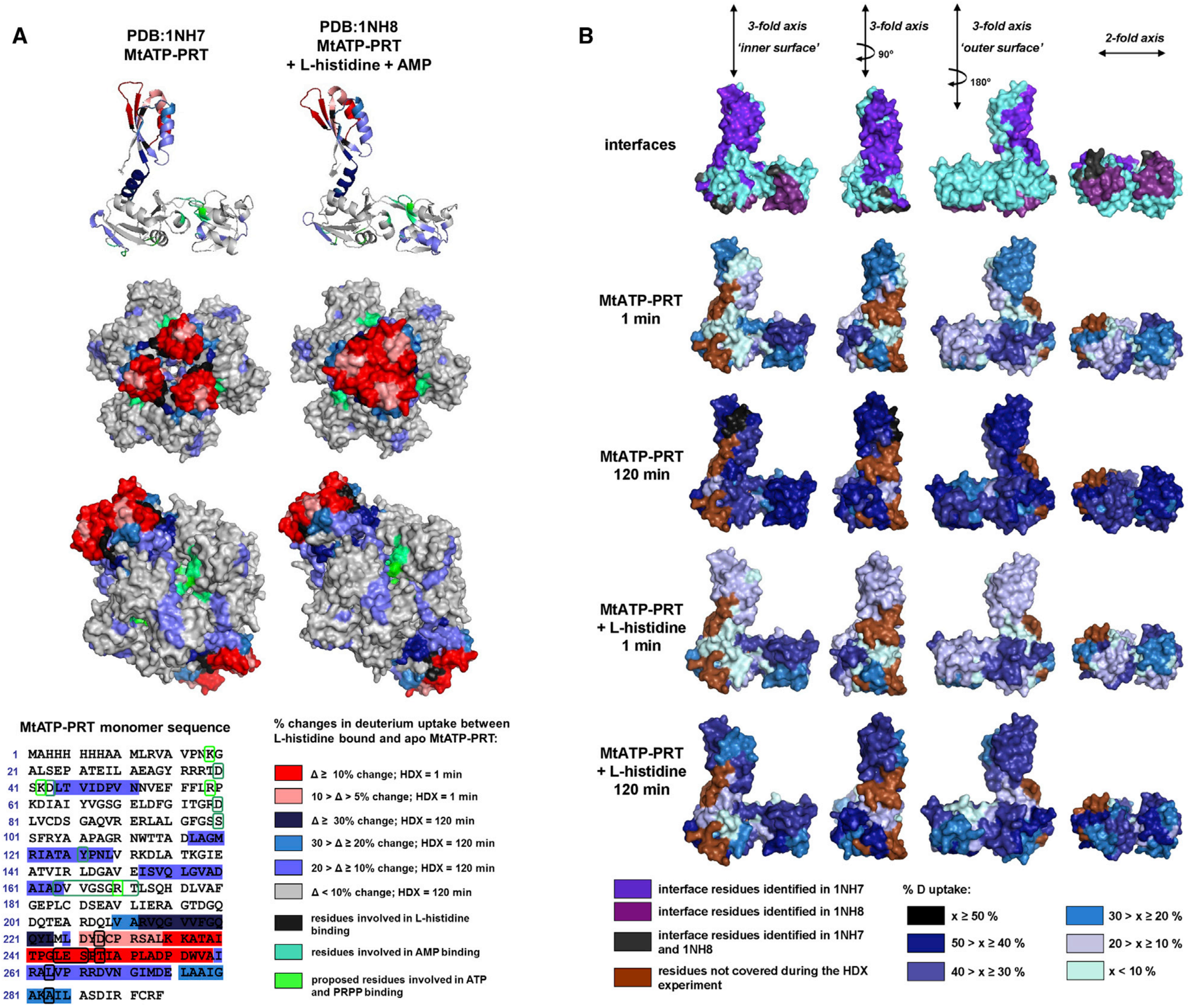


**Figure 4. HDX-MS Data Providing Local Insights into Conformational Changes in MtATP-PRT**

(A) Percentage differences in deuterium exchange visualized on the PDB crystal structures: PDB: 1NH7 and 1NH8 and the MtATP-PRT monomer sequence. Residues with significant changes in HDX on short timescale are highlighted in red; residues where changes occur on a longer timescale (120 min) are highlighted in shades of blue. Residues involved in direct interactions with L-histidine, residues involved in binding of AMP, and residues proposed to be involved in ATP and PRPP binding as reported by Cho et al. (2003). are shown in black, teal green, and lime green, respectively.

(B) Percentage D exchange of MtATP-PRT in the presence and in the absence of L-histidine at pH 6.8 after 1 min and 120 min of exposure to deuterated buffer represented on MtATP-PRT monomer (PDB: 1NH7) viewed from different angles. The top row highlights interface residues based on PDB: 1NH7 (violet) and PDB: 1NH8 (purple). The increasing intensity of blue correlates with increase in D uptake. Residues not covered during the HDX experiment are highlighted in brown. See also Figures S5 and S6; Tables S2 and S3A–S3E.

Nambi et al., 2012). At pH 9.0, the differences in D uptake upon L-histidine are more subtle (Figure S6B). A minor variation in D uptake is noted around domains I and III (peptides 63–85), indicating some binding is occurring; however, no significant changes on longer timescales associated with conformational changes are observed around the active site. The datasets obtained under different buffer conditions cannot be compared with each other due to the pH dependence of the intrinsic HDX rate (Bai et al., 1993; Hui et al., 2005). Nevertheless, data obtained under identical buffer conditions serve to map regions of ligand binding and conformational changes (Chalmers et al., 2011; Zhang et al., 2013).

## Probing the Protein Complex Interfaces with HDX-MS

The percentage of absolute D exchange was calculated for MtATP-PRT in the presence and absence of L-histidine at pH 6.8 at 1 min and 120 min exchange time points and was mapped onto the MtATP-PRT monomer structure. The MtATP-PRT monomer is presented in Figure 4B at various angles: three snapshots along the 3-fold symmetry axis of the hexamer and one along the 2-fold symmetry plane of the hexamer. An increased percentage of absolute D exchange is visualized with increasing intensity of blue. In addition, the interface residues were identified using PISA (Krissinel, 2010; Krissinel and Henrick, 2007) based on the PDB: 1NH7 (violet) and

PDB: 1NH8 (purple) crystal structures as shown in the top row of Figure 4B. Some of the crucial residues at the subunit interfaces were not covered by peptides resulting from pepsin digestion (brown). Nevertheless, it is seen that a lower amount of D is incorporated into the surface residues facing the intra-subunit cavity, i.e., inner surface as opposed to the residues on the outer surface, within the first minute of HDX. Such pattern is observed both in the presence and absence of L-histidine, and is supportive of the mechanism proposed by Pedreño et al. (2012), which proposes MtATP-PRT as a functional hexamer where conformational changes have to be invoked to explain the allosteric inhibition instead of changes in oligomeric state (Figure 1E). Moreover, upon addition of L-histidine, the percentage of absolute D exchange on a long timescale at the inner surface is lower in comparison with D exchange in the absence of ligand, further supporting conformational rearrangement to a more compact structure and reducing solvent penetration of the intra-subunit cavity.

## Conclusions

A set of MS-based techniques has been employed to investigate the allosteric inhibition mechanism of MtATP-PRT, a 190 kDa homo-hexameric enzyme catalyzing the first step of the biosynthesis of L-histidine in *Mycobacterium tuberculosis*. Native MS reveals MtATP-PRT to exist in a hexameric state under physiological conditions. Although the binding stoichiometry could not be conclusively determined, in-source dissociation MS experiments suggest that the binding of the first four L-histidine molecules may have different affinity from that of the subsequent two.

Conformational changes induced by L-histidine binding and the influence of pH were probed with IM-MS, HDX-MS, and AUC. Results from all three techniques support the occurrence of subtle conformational changes upon ligand binding at pH 6.8. Linear drift-tube IM-MS experiments showed a decrease in the mean $^{DT}CCS_{He}$ from 76.6 $nm^2$ to 73.5 $nm^2$ (for the [28+] charge state), and this change was also confirmed by the theoretical CCS calculated from the available crystal structures. No such changes in conformation were found to take place at pH 9.0. Sedimentation velocity analysis confirmed the conformational tightening upon ligand binding observed in vacuo at pH 6.8.

Furthermore, HDX-MS was used for mapping of the conformational changes, and the results are in agreement with X-ray crystallography data. Changes in the deuterium exchange between apo MtATP-PRT and L-histidine-bound MtATP-PRT occurring on a short timescale were found within domain III and are associated with L-histidine binding to the allosteric site. Changes occurring on longer timescales related to conformational changes induced by ligand binding were identified around the active site and near the residues involved in AMP binding and residues proposed to be involved in binding of ATP and PRPP substrates. The collected data support the mechanism proposed by Pedreño et al. (2012), which proposes that MtATP-PRT exists as a functional hexamer, refuting the hypothesis of changes in the oligomeric state upon allosteric inhibition. Definition of the exact mechanism of allosteric inhibition is key to the rational development of improved compounds that might be able to control flux through biochemical pathways to either kill cells, in the context of therapeutics, or maximize metabolite production, in the context of synthetic biology. Native MS approaches as used in this study can be a next generation of tools to rapidly and unambiguously access allosteric inhibition mechanisms.

## STAR★METHODS

Detailed methods are provided in the online version of this paper and include the following:

- KEY RESOURCES TABLE
- CONTACT FOR REAGENT AND RESOURCE SHARING
- METHOD DETAILS
  - Protein Expression and Purification
  - Measurement of Enzymatic Activity
  - Sample Preparation
  - Native Mass Spectrometry
  - Drift Tube IM-MS
  - Determination of $^{DT}CCS_{He}$
  - TWIMS-MS
  - Determination of Theoretical CCS
  - Bead Models
  - Hydrogen Deuterium Exchange MS
  - Analytical Ultracentrifugation
- QUANTIFICATION AND STATISTICAL ANALYSIS
  - Construction of the Binding Plot

## SUPPLEMENTAL INFORMATION

Supplemental Information includes six figures and three tables and can be found with this article online at http://dx.doi.org/10.1016/j.str.2017.03.005.

## AUTHOR CONTRIBUTIONS

K.J.P. performed all the direct infusion mass spectrometry experiments, the data analysis, and wrote the initial draft of the manuscript. V.O. assisted at the early stage, and M.P. did the computation analysis for CCS data from the crystal structures. R.J.B., R.J.T., and R.A.G. assisted with the HDX work. T.A.J. performed the AUC and data analysis. J.P.P. and G.L.-M. performed the protein expression. K.J.P., L.P.S.de.C., and P.E.B. designed the study and P.E.B. finalized the manuscript. All authors contributed to robust discussions regarding the data and how to interpret it.

## ACKNOWLEDGMENTS

This work was supported by the Medical Research Council through the award of an Industrial Case Studentship to K.J.P. in collaboration with UCB, and by the BBSRC grants BB/L015048/1, and BB/H013636/1, and the BBSRC/EPSRC-funded Manchester Synthetic Biology Research Center, SYNBIOCHEM (BB/M017702/1). We also thank the Francis Crick Institute, which receives its core funding from Cancer Research UK, the Medical Research Council, and the Wellcome Trust. The authors are very grateful to the British Mass Spectrometry Society for a grant that allowed us to purchase our nanospray tip puller still going strong after 13 years of pulling.

Received: October 12, 2016
Revised: January 9, 2017
Accepted: March 9, 2017
Published: April 6, 2017

## REFERENCES

Bai, Y.W., Milne, J.S., Mayne, L., and Englander, S.W. (1993). Primary structure effects on peptide group hydrogen-exchange. Proteins *17*, 75–86.

Beveridge, R., Migas, L.G., Payne, K.A.P., Scrutton, N.S., Leys, D., and Barran, P.E. (2016). Mass spectrometry locates local and allosteric conformational changes that occur on cofactor binding. Nat. Commun. *7*, 12163.

Brautigam, C.A. (2015). Calculations and publication-quality illustrations for analytical ultracentrifugation data. In Analytical Ultracentrifugation, J.L. Cole, ed. (Elsevier), pp. 109–133.

Brookes, E., Demeler, B., Rosano, C., and Rocco, M. (2010). The implementation of SOMO (SOlution MOdeller) in the UltraScan analytical ultracentrifugation data analysis suite: enhanced capabilities allow the reliable hydrodynamic modeling of virtually any kind of biomacromolecule. Eur. Biophys. J. *39*, 423–435.

Case, D.A., Darden, T.A., Cheatham, T.E., III, Simmerling, C.L., Wang, J., Duke, R.E., Luo, R., Walker, R.C., Zhang, W., Merz, K.M., et al. (2010). Amber 11 (University of California, San Francisco).

Chalmers, M.J., Busby, S.A., Pascal, B.D., West, G.M., and Griffin, P.R. (2011). Differential hydrogen/deuterium exchange mass spectrometry analysis of protein-ligand interactions. Expert Rev. Proteomics *8*, 43–59.

Cho, Y., Sharma, V., and Sacchettini, J.C. (2003). Crystal structure of ATP phosphoribosyltransferase from *Mycobacterium tuberculosis*. J. Biol. Chem. *278*, 8333–8339.

Coval, M.L. (1970). Analysis of Hill interaction coefficients and invalidity of Kwon and Brown equation. J. Biol. Chem. *245*, 6335–6336.

Demeler, B. (2005). UltraScan - a comprehensive data analysis software package for analytical ultracentrifugation experiments. In Analytical Ultracentrifugation: Techniques and Methods, E. Scott, S.E. Harding, and A. Rowe, eds. (Royal Society of Chemistry), pp. 210–230.

Dyachenko, A., Gruber, R., Shimon, L., Horovitz, A., and Sharon, M. (2013). Allosteric mechanisms can be distinguished using structural mass spectrometry. Proc. Natl. Acad. Sci. USA *110*, 7235–7239.

Edfeldt, F.N.B., Folmer, R.H.A., and Breeze, A.L. (2011). Fragment screening to predict druggability (ligandability) and lead discovery success. Drug Discov. Today *16*, 284–287.

Hardy, J.A., and Wells, J.A. (2004). Searching for new allosteric sites in enzymes. Curr. Opin. Struct. Biol. *14*, 706–715.

Heck, H.D.A. (1971). Statistical theory of cooperative binding to proteins - Hill equation and binding potential. J. Am. Chem. Soc. *93*, 23–29.

Hui, X., Hoerner, J.K., Eyles, S.J., Dobo, A., Voigtman, E., Mel'Cuk, A.I., and Kaltashov, I.A. (2005). Mapping protein energy landscapes with amide hydrogen exchange and mass spectrometry: I. A generalized model for a two-state protein and comparison with experiment. Protein Sci. *14*, 543–557.

Iacob, R.E., Zhang, J., Gray, N.S., and Engen, J.R. (2011). Allosteric interactions between the myristate- and ATP-site of the Abl kinase. PLoS One *6*, e15929.

Jurneczko, E., and Barran, P.E. (2011). How useful is ion mobility mass spectrometry for structural biology? The relationship between protein crystal structures and their collision cross sections in the gas phase. Analyst *136*, 20–28.

Konijnenberg, A., Butterer, A., and Sobott, F. (2013). Native ion mobility-mass spectrometry and related methods in structural biology. Biochim. Biophys. Acta *1834*, 1239–1256.

Krishnamurthy, S., Moorthy, B.S., Lin, L., and Anand, G.S. (2013). Dynamics of phosphodiesterase-induced cAMP dissociation from A protein kinase A: capturing transient ternary complexes by HDXMS. Biochim. Biophys. Acta *1834*, 1215–1221.

Krissinel, E. (2010). Crystal contacts as nature's docking solutions. J. Comput. Chem. *31*, 133–143.

Krissinel, E., and Henrick, K. (2007). Inference of macromolecular assemblies from crystalline state. J. Mol. Biol. *372*, 774–797.

Lindorff-Larsen, K., Piana, S., Palmo, K., Maragakis, P., Klepeis, J.L., Dror, R.O., and Shaw, D.E. (2010). Improved side-chain torsion potentials for the Amber ff99SB protein force field. Proteins *78*, 1950–1958.

Marcoux, J., and Robinson, C.V. (2013). Twenty years of gas phase structural biology. Structure *21*, 1541–1550.

Martin, R.G. (1963). First enzyme in histidine biosynthesis: the nature of feedback inhibition by histidine. J. Biol. Chem. *238*, 257–268.

McCullough, B.J., Kalapothakis, J., Eastwood, H., Kemper, P., MacMillan, D., Taylor, K., Dorin, J., and Barran, P.E. (2008). Development of an ion mobility quadrupole time of flight mass spectrometer. Anal. Chem. *80*, 6336–6344.

Nambi, S., Badireddy, S., Visweswariah, S.S., and Anand, G.S. (2012). Cyclic AMP-induced conformational changes in mycobacterial protein acetyltransferases. J. Biol. Chem. *287*, 18115–18129.

Pacholarz, K.J., Garlish, R.A., Taylor, R.J., and Barran, P.E. (2012). Mass spectrometry based tools to investigate protein-ligand interactions for drug discovery. Chem. Soc. Rev. *41*, 4335–4355.

Pacholarz, K.J., Porrini, M., Garlish, R.A., Burnley, R.J., Taylor, R.J., Henry, A.J., and Barran, P.E. (2014). Dynamics of intact immunoglobulin G explored by drift-tube ion-mobility mass spectrometry and molecular modeling. Angew. Chem. Int. Ed *53*, 7765–7769.

Pedreño, S., Pisco, J.P., Larrouy-Maumus, G., Kelly, G., and de Carvalho, L.P.S. (2012). Mechanism of feedback allosteric inhibition of ATP phosphoribosyltransferase. Biochemistry *51*, 8027–8038.

Pukala, T.L., Ruotolo, B.T., Zhou, M., Politis, A., Stefanescu, R., Leary, J.A., and Robinson, C.V. (2009). Subunit architecture of multiprotein assemblies determined using restraints from gas-phase measurements. Structure *17*, 1235–1243.

Schuck, P. (2000). Size-distribution analysis of macromolecules by sedimentation velocity ultracentrifugation and Lamm equation modeling. Biophys. J. *78*, 1606–1619.

Shvartsburg, A.A., Schatz, G.C., and Jarrold, M.F. (1998). Mobilities of carbon cluster ions: critical importance of the molecular attractive potential. J. Chem. Phys. *108*, 2416–2423.

Skinner, J.J., Lim, W.K., Bedard, S., Black, B.E., and Englander, S.W. (2012). Protein dynamics viewed by hydrogen exchange. Protein Sci. *21*, 996–1005.

Surade, S., and Blundell, T.L. (2012). Structural biology and drug discovery of difficult targets: the limits of ligandability. Chem. Biol. *19*, 42–50.

Voll, M.J., Appella, E., and Martin, R.G. (1967). Purification and composition studies of phosphoribosyl-adenosine triphosphate-pyrophosphate phosphoribosyltransferase first enzyme of histidine biosynthesis. J. Biol. Chem. *242*, 1760–1767.

Zhang, J., Adrian, F.J., Jahnke, W., Cowan-Jacob, S.W., Li, A.G., Iacob, R.E., Sim, T., Powers, J., Dierks, C., Sun, F., et al. (2010). Targeting Bcr-Abl by combining allosteric with ATP-binding-site inhibitors. Nature *463*, 501–506.

Zhang, Q., Chen, J., Kuwajima, K., Zhang, H.-M., Xian, F., Young, N.L., and Marshall, A.G. (2013). Nucleotide-induced conformational changes of tetradecameric GroEL mapped by H/D exchange monitored by FT-ICR mass spectrometry. Sci. Rep. *3*, 1247.

# STAR★METHODS

## KEY RESOURCES TABLE

| REAGENT or RESOURCE | SOURCE | IDENTIFIER |
|---|---|---|
| Chemicals, Peptides, and Recombinant Proteins | | |
| Acetonitrile | Sigma | Cat#CN34851 |
| Adenosine triphosphate | Sigma | Cat#A2383 |
| Ammonia solution | VWR International Ltd | Cat#470300-172 |
| Ammonium acetate | Fisher Scientific | Cat#A637-500 |
| CHES buffer | Acros Organics | Cat#208185000 |
| Complete EDTA-free protease inhibitor cocktail | Sigma (Roche) | Cat#COEDTAF-RO |
| Deuterium oxide, 99.9 atom % D | Sigma | Cat#151882 |
| Formic acid | Sigma | Cat#FX0440 |
| HEPES buffer | Acros Organics | Cat#172571000 |
| GluFib peptide | Waters | Cat#700004729 |
| Guanidine hydrochloride | Sigma | Cat#G4505 |
| Hydrochloric acid | VWR International Ltd | Cat#470301-206 |
| Imidazole | Acros Organics | Cat#301870025 |
| L-histidine | Acros Organics | Cat#166150250 |
| Lysozyme | Sigma | Cat#L6876 |
| Magnesium chloride | Sigma | Cat#M8266 |
| Phosphoribosyl pyrophosphate | Sigma | Cat#P8296 |
| Potassium chloride | Fisher Scientific | Cat#P/4240/53 |
| Potassium phosphate dibasic | Sigma | Cat#P8281 |
| Potassium phosphate monobasic | Sigma | Cat#P5655 |
| Pyrophosphatase | de Carvalho group | N/A |
| Sodium chloride | Fisher Scientific | Cat#S/3160/60 |
| TAPS buffer | Acros Organics | Cat#327801000 |
| Triethanolamine | Acros Organics | Cat#139560010 |
| Triethylamine acetate buffer | Fluka | Cat#90357 |
| Tris(2-carboxyethyl)phosphine hydrochloride | Sigma | Cat#C4706 |
| Tris-HCl (Trizma base) | Sigma | Cat#93362 |
| WT MtATP-Phosphoribosyltransferase | de Carvalho group | N/A |
| Deposited Data | | |
| MtATP-PRT x-ray structure | (Cho et al., 2003) | PDB: 1NH7 |
| Mt-ATP-PRT – L-histidine complex x-ray structure | (Cho et al., 2003) | PDB: 1NH8 |
| Experimental Models: Cell Lines | | |
| BL21(DE3)pLysS (*pJ411::hisG*) cells | Millipore | Cat#6941 |
| Recombinant DNA | | |
| H37Rv *pJ411* plasmid | made by DNA2.0 | N/A |
| Software and Algorithms | | |
| Amber11 | (Case et al., 2010) | http://ambermd.org |
| Amber99SB-ILDN force field | (Lindorff-Larsen et al., 2010) | http://ambermd.org |
| DynamX Data Analysis v2.0 | Waters | http://www.waters.com |
| GUSSI | (Brautigam, 2015) | http://biophysics.swmed.edu/MBR/software.html |
| MassLynx | Waters | http://www.waters.com |

(*Continued on next page*)

*Continued*

| REAGENT or RESOURCE | SOURCE | IDENTIFIER |
|---|---|---|
| MOBCAL | (Shvartsburg et al., 1998) | http://www.indiana.edu/~nano/software/ |
| Origin 8.5 | OriginLab | http://www.originlab.com |
| PyMOL Molecular Graphics System, V1.5.0.4 | Schrödinger | http://www.pymol.com |
| Sedfit | (Schuck, 2000) | https://sedfitsedphat.nibib.nih.gov/software/default.aspx |
| SOMO | (Brookes et al., 2010) | http://www.somo.uthscsa.edu/ |
| Ultrascan v9.9 | (Demeler, 2005) | http://www.ultrascan.uthscsa.edu |
| Other | | |
| C18 column (BEH C18, 100 × 1.0 mm, 1.7 μM) | Waters | Cat#186002346 |
| Deactivated glass screw neck vials | Waters | Cat#186000327c |
| Glass capillaries (ID 0.9 mm, OD 1.2 mm) | World Precision Instruments | Cat#TW120-4 |
| Micro Bio-Spin 6 Columns, Tris | Bio-Rad | Cat#7326222 |
| Micro-loading tips | Eppendorf | Cat#5242956.003 |
| Pepsin column (3 cm Poroszyme immobilized) | Applied Biosystems | Cat#2313100 |
| Platinum wire | GoodFellow | Cat#LS388670/1 |
| Superdex 200 10/300 gel filtration column | Superdex | Cat#17-5175-01 |
| VanGuard C18 trap column | Waters | Cat#186003975 |

## CONTACT FOR REAGENT AND RESOURCE SHARING

Further information and requests for reagents should be directed to and will be fulfilled by the Lead Contact, Perdita E. Barran (perdita.barran@manchester.ac.uk).

## METHOD DETAILS

### Protein Expression and Purification

The *rv2121c* (*hisG*) gene sequence from *M. tuberculosis* H37Rv was codon adapted to *E. coli*, and its nucleotide sequence was synthetically prepared and ligated into the *pJ411* plasmid (DNA 2.0). DNA sequence was confirmed by sequencing. This construct contained a noncleavable N-terminal hexahistidine tag to facilitate purification. The N-terminal hexahistidine tag was shown not to affect the structure or activity of MtATP-PRT. During MtATP-PRT purification, all steps were performed at 4 °C. Frozen BL21(DE3)pLysS (*pJ411*::*hisG*) cells were thawed on ice, and lysed by sonication, in the presence of buffer A [20 mM triethanolamine (TEA) (pH 7.8), 300 mM NaCl, and 50 mM imidazole] containing lysozyme and complete EDTA-free protease inhibitor cocktail. Soluble protein was obtained by centrifugation at 25000g for 30 min. The soluble fraction was loaded into a 50 mL Ni-NTA column and the protein separated by a gradient using buffer B [20 mM TEA (pH 7.8), 300 mM NaCl, and 500 mM imidazole]. Peak fractions were analyzed by SDS−PAGE. Fractions containing only MtATP-PRT were pooled together, concentrated, dialyzed in 20 mM TEA (pH 7.8), and stored at −80 °C after being flash-frozen in liquid nitrogen.

### Measurement of Enzymatic Activity

Steady-state kinetic assays were conducted with a Shimadzu UV-2550 spectrophotometer equipped with dual-beam optics and a Peltier system for temperature control. Initial velocities for the forward reaction of MtATP-PRT were measured by following the formation of PR-ATP ($\varepsilon_{290}$ = 3,600 $M^{-1}$ $cm^{-1}$), in the presence of pyrophosphatase (PPase). PPase is essential for this assay, as the equilibrium constant lies towards formation of ATP and PRPP. A typical reaction mixture contained 50 mM Tris-HCl (pH 8.5), 7 mM $MgCl_2$, 200 mM KCl, 3 mM ATP, 1.5 mM PRPP, 600 nM PPase and 450 nM MtATP-PRT. All kinetic assays were conducted at 25 °C ± 0.2 °C. To determine the effect of pH on MtATP-PRT activity, the maximum velocity was measured as described above at different pH values ranging from 7.75 to 10.00 [in 50 mM HEPES (7.75 to 8.00), TAPS (8.25 to 8.75) and CHES (9.00 to 10.00) buffers].

### Sample Preparation

On the day of analysis, buffer was exchanged to 100 mM ammonium acetate buffer (Fisher Scientific, Loughborough, UK) of specified pH, using micro Bio-Spin Chromatography columns (Micro Bio-Spin 6 Columns, Tris) following the instructions specified by the manufacturer. The desalting procedure was performed four to five times to achieve desired sample quality. pH of the buffer was

adjusted with hydrochloric acid or ammonia supplied by VWR International Ltd (UK). Solution pH readings were taken using a pH meter (Jenway 3305). High purity water was obtained from an Arium 611 water purification unit (Sartorius, Göttingen, Germany) fitted with a 0.2 μm filter. Charge reduction experiments were carried out by addition of 10% (v) triethylamine acetate buffer (TEAA) (Fluka, Steinheim, Switzerland) of ethylenediammonium diacetate (EDDA) prior to MS analysis.

## Native Mass Spectrometry

Mass spectra were recorded on a Q-ToF mass spectrometer (Ultima API US, Waters, Manchester, UK) modified for high mass molecules. NanoESI capillaries were prepared in-house from thin-walled glass capillaries (inner diameter 0.9 mm, outer diameter 1.2 mm, World Precision Instruments, Stevenage, UK) using a Flaming/Brown P-97 micropipette puller (Sutter Instrument Company, Novato, CA, USA). These were then filled with sample using micro-loading tips (Eppendorf, Hamburg, Germany). A positive voltage was applied to the solution via a platinum wire inserted into the capillary (GoodFellow, Huntingdon, UK). The following experimental parameters were used (positive ion mode): capillary voltage, ~1.5 kV; cone gas, 0 L/hr; sample cone, 50-200 V; extractor cone, 5 V; collision energy, 5.0 V; MCP 2400 V; source temperature: 30 °C; source pressure, 8.5 x $10^{-3}$ bar ion transfer stage pressure, ~4.0 x $10^{-3}$ bar; quadruple analyzer pressure, 1.4 x $10^{-5}$ bar; TOF analyzer pressure, 1.7 x $10^{-7}$ bar. External calibration of the spectra was achieved using solutions of cesium iodide (2 mg/mL in 50:50 water:isopropanol). Data were acquired and processed with MassLynx software (Waters, Manchester, UK).

## Drift Tube IM-MS

The IM-MS data were acquired on the MoQToF, quadrupole time-of-flight mass spectrometer modified in-house to include 5.1 cm drift cell (McCullough et al., 2008). Same in-house made nano-capillaries were used as described above. The source temperature was set to 30 °C. The nano-ESI capillary voltage of 1.3-1.7 kV was used. Experiments were performed at ~3.75 Torr helium pressure (measured using a baratron, MKS Instruments) and ~300 K cell temperature. A continuous beam of ions is produced by nanoESI source and a DC voltage on 'top hat' ion gate lens traps ions is raised and ions are pulsed out of hexapole in "ion packet" (100 μs width, ~ 36 Hz). The frequency of this pulse was set using a signal generator and is dependent on the ToF pusher period which in turn is dependent on *m/z* of analyzed species (165 μs). The injection energy, a potential difference between the first transfer hexapole and the drift cell, used was between 30 V and 34 V. The drift voltage across the cell was varied to obtain ion mobility data by decreasing the cell body potential from 60 to 20 V. Ion arrival time distributions were recorded by synchronization of the release of ions into the drift cell with mass spectral acquisition. The rotationally-averaged collision cross-sections ($^{DT}CCS_{He}$) were determined from a plot of arrival time versus *P* / *T* as described in a section below. Data were processed using Microsoft Excel and Origin 8.5 (Origin Lab).

## Determination of $^{DT}CCS_{He}$

In the IM-MS experiment, a total arrival time distribution (ATD), corresponding to one mobility separation, is generated every 200 MS scans. Spectra were acquired for at least fifteen pulses per each drift voltage and summed. Pressure and temperature is recorded before and after each set of scans and averaged for further calculations. The average scan number for each conformer is calculated by fitting a Gaussian distribution and determining the midpoint with help of Origin graphing software. Average arrival time ($t_a$) is obtained by multiplication of the average scan number by the MS pusher period used in the experiment. Average arrival time ($t_a$) corresponds to the time the ion takes to travel through the drift cell (the drift time, $t_d$) plus the time taken to reach the detector after exiting the drift cell (the dead time, $t_0$). Next, the arrival time ($t_a$) is plotted as function of P/V and a linear fit to the data-points can be made. The intercept of this fitted line provides the instrumental 'dead time' ($t_0$), and the slope of the line is inversely proportional to the reduced mobility ($K_0$) in the following way:

$$t_d = t_a - t_0 = \frac{L^2 T_0 P}{K_0 T P_0 V} \qquad \text{(Equation 1)}$$

where *L* is the length of the tube, *V* is the drift voltage, *P* is pressure, $P_0$ is 760 Torr, *T* is temperature and $T_0$ is 273.15 K. The rotationally-averaged collision cross section (Ω) can be then calculated from $K_0$ knowing that:

$$\Omega = \frac{3ze}{16N}\left(\frac{2\pi}{\mu k_B T}\right)^{1/2}\frac{1}{K_0} \qquad \text{(Equation 2)}$$

where *N* is the number density of the buffer gas, $\mu$ is the reduced mass of the analyte and the buffer gas and $k_B$ is the Boltzmann constant. Each complete IM-MS experiment is performed three times and the mean collision cross-sections are calculated for each charge state.

## TWIMS-MS

The IM-MS data were acquired on the Synapt G2S HDMS (Waters, Manchester, UK) at the Waters Corporation, Floats Road, Manchester, UK. In-house made nano-capillaries were used as described previously. The samples were prepared in 100 mM ammonium acetate buffer at pH 6.8 and pH 9.0. Gentle conditions were applied to preserve the native-like structure: capillary voltage 1.6 kV, sampling cone 99 V, source temperature 20 °C, trap collision energy 5.6 V, and transfer collision energy 2 V. The pressure of the

backing region was 8.3 mbar. For IM, the helium cell and the IMS gas flows were 180 and 90 mL/min, respectively, the IMS wave velocity was 617 m/s, and the IMS wave height was 40 V. Nitrogen was the carrier gas. Data were acquired and processed with MassLynx software (Waters, Manchester, UK).

### Determination of Theoretical CCS

The input coordinates files were those taken from the crystallographic structures, PDB identifiers 1NH7 and 1NH8 for apo MtATP-PRT and Mt-ATP-PRT – L-histidine complex, respectively. After adding hydrogen atoms, the x-ray crystal structures were minimized *in vacuo* with the sander module of Amber11 (Case et al., 2010), implementing a radial cut-off of 999 Å and Amber99SB-ILDN force (Lindorff-Larsen et al., 2010). The rotationally averaged CCS were calculated with the trajectory method of MOBCAL code (Shvartsburg et al., 1998) appropriately modified to handle large systems such as MtATP-PRT studied here.

### Bead Models

Bead models were constructed using the crystal coordinates 1NH7 (Apo) and 1NH8 (Holo) and created using the solution bead modelling software SOMO (Brookes et al., 2010) within the Ultrascan software V9.9 (Demeler, 2005).

### Hydrogen Deuterium Exchange MS

HDX-MS analysis was achieved using a Waters HDX module with nanoAcquity UPLC and Synapt G2 mass spectrometer. Sample handling steps were performed by a LEAP-PAL robotics system. 30 μM protein solutions were diluted 20 fold into 10 mM phosphate in either $H_2O$ or $D_2O$, pH/pD 7, and the mixture incubated at 20 °C for 0 minutes ($H_2O$), or 1, 10, 30 or 120 minutes ($D_2O$), before the quench step. HDX quenching was achieved by mixing the reaction solution 1:1 with cooled 3.4 M Gdn-HCl, 500 mM TCEP, 200 mM phosphate (pH 2.5, 0 °C). 37.5 pmol was injected into the HDX module (0 °C), and washed over the pepsin column (Applied Biosystems, 3 cm Poroszyme immobilised pepsin column) with 0.1% HCOOH in $H_2O$, pH 2.5, at 200 $\mu L.min^{-1}$. Resulting peptides were trapped on a VanGuard C18 trap column. Peptide separation was achieved on a C18 column (BEH C18, 100 × 1.0 mm, 1.7 μM) with the following gradient: 0 min: 8% B, 7 min: 32% B, 8 min: 85% B (mobile phase A: 0.1% HCOOH in $H_2O$, pH 2.5; mobile phase B: 0.1% HCOOH in MeCN). The mass spectrometer was operated in ToF only mode, with $MS^e$ data acquisition (trap collision energy ramp 14-35V). Calibration was achieved from the MS/MS spectrum of GluFib peptide. PLGS v2.5 and DynamX Data Analysis v2.0 software (Waters Corporation, Manchester, UK) were used for data analysis. The deuterium update protein maps were created in the PyMOL Molecular Graphics System, V1.5.0.4 (Schrödinger, LLC., Portland, OR, USA). The mass increase was calculated for each identified peptide at all four labelling time points and compared across experimental conditions *i.e.* ligand presence and pH. The percentage deuterium exchange has been calculated by dividing the amount of measured deuterium update in Da over the maximum theoretical deuterium update in Da and multiplied by 100.

### Analytical Ultracentrifugation

His-tagged MtATP-PRT was purified and buffer exchanged by running peak fractions twice on a Superdex200 10/300 gel filtration column attached to a multi-angle laser light scattering system (MALLS) in ammonium acetate buffer at either pH 6.8 or pH 9.0. The peak fractions for each pH separation corresponding to hexameric protein were pooled and the concentration assayed using a spectrometer reading at 280 nm and a molar extinction coefficient of 20190 $M^{-1}$ $cm^{-1}$ for monomeric protein. The final concentrations were ~40 μM (hexamer) and to this 12-fold molar excess L-histidine from a 50 mM stock in HCl was added to one 0.5 ml fraction for both pH 6.8 and pH 9.0. Samples were loaded (400 μl) into 2-sector sedimentation velocity cells with sapphire windows and loaded into a XL-I ultracentrifuge (Beckman). Sedimentation was monitored every 1 minute at 45,000 rpm, 20 °C using interference optics and data analysed using the direct boundary modelling software Sedfit (Schuck, 2000) and represented using GUSSI (Brautigam, 2015).

## QUANTIFICATION AND STATISTICAL ANALYSIS

### Construction of the Binding Plot

Relative spectral intensity of the [23+] and [27+] charge states was plotted as a function of number of titrated L-histidine equivalents with respect to MtATP-PRT hexamer. The following were used: [23+]/([23+]+[27+]) and [27+]/([23+]+[27+]) for [23+] and [27+] charge state respectively, to determine the relative intensity at each L-histidine equivalent point presented in Figure 3B.

Structure, Volume *25*

## Supplemental Information

# Hybrid Mass Spectrometry Approaches to Determine How L-Histidine Feedback Regulates the Enzyzme MtATP-Phosphoribosyltransferase

**Kamila J. Pacholarz, Rebecca J. Burnley, Thomas A. Jowitt, Victoria Ordsmith, João Pedro Pisco, Massimiliano Porrini, Gérald Larrouy-Maumus, Rachel A. Garlish, Richard J. Taylor, Luiz Pedro Sório de Carvalho, and Perdita E. Barran**

# Supplemental Information

# Hybrid Mass Spectrometry Approaches to Determine how L-Histidine Feedback Regulates the Enzyme MtATP-Phosphoribosyltransferase

Kamila J. Pacholarz[1,2], Rebecca J. Burnley[3], Thomas A. Jowitt[4], Victoria Ordsmith [2], João Pedro Pisco[5], Massimiliano Porrini[6], Gérald Larrouy-Maumus[5,$], Rachel A. Garlish[3], Richard J. Taylor[3], Luiz Pedro Sório de Carvalho[5*], Perdita E. Barran[1 *]

[1] Michael Barber Centre for Collaborative Mass Spectrometry, Manchester Institute of Biotechnology, 131 Princess Street, M1 7DN & School of Chemistry, University of Manchester, Oxford Road, M13 9PL, Manchester, UK
[2]School of Chemistry, University of Edinburgh, David Brewster Road, EH9 3FJ, Edinburgh, UK
[3] Physical and Analytical Sciences Group, UCB, 216 Bath Road, SL1 4EN, Slough, UK
[4] The Biomolecular Analysis Facility, Faculty of Life Sciences, University of Manchester, Oxford Road, M13 9PT, Manchester, UK
[5] Mycobacterial Metabolism and Antibiotic Research Laboratory, The Francis Crick Institute, 1 Midland Road, NW1 1AT, London, UK
[6] ARNA Laboratory, Inserm U1212/CNRS UMR 5320/University of Bordeaux, France
[1] Lead Contact, perdita.barran@manchester.ac.uk

**Supporting Data**

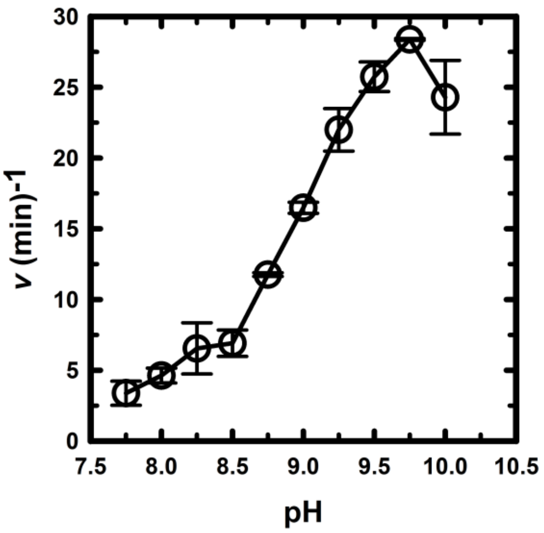


**Figure S1. Related to Figure 1. MtATP-PRT activity pH dependence. Below pH 7.75, despite very slow, MtATP-PRT is still active.**

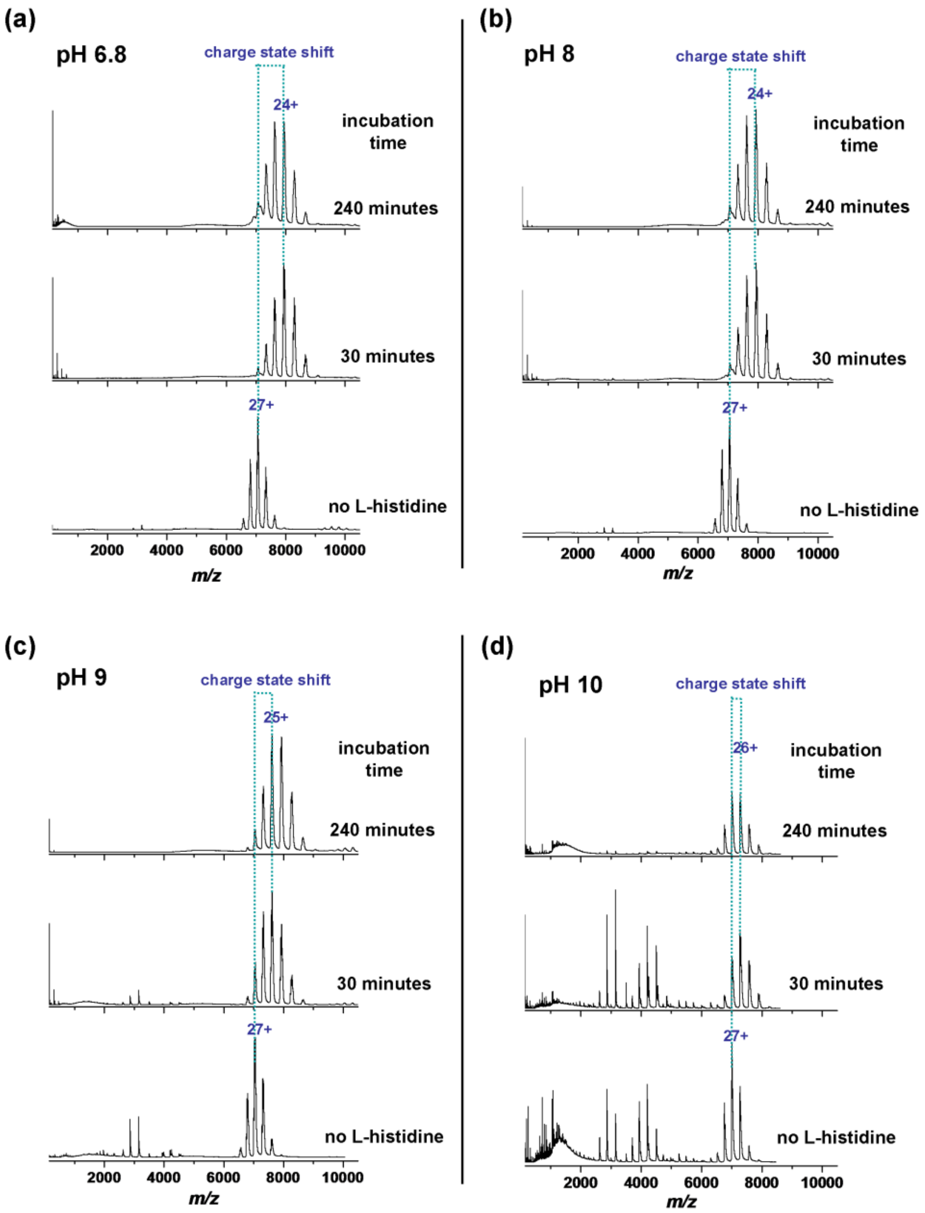


**Figure S2. Related to Figure 2. nESI mass spectra of 20 µM apo MtATP-PRT (hexamer concentration) and MtATP-PRT incubated with 12 equivalents of L-histidine for 30 minutes and 240 minutes in 100 mM ammonium acetate at (a) pH 6.8, (b) pH 8, (c) pH 9 and (d) pH 10, acquired on Ultima API US Q-ToF mass spectrometer (at cone voltage 268 V). Efforts to shift charge state distribution (CSD) to a higher *m/z* range by addition of charge reducing agents such as TEAA and EDDA (*5*) did not improve resolution sufficiently to determine the binding stoichiometry.**

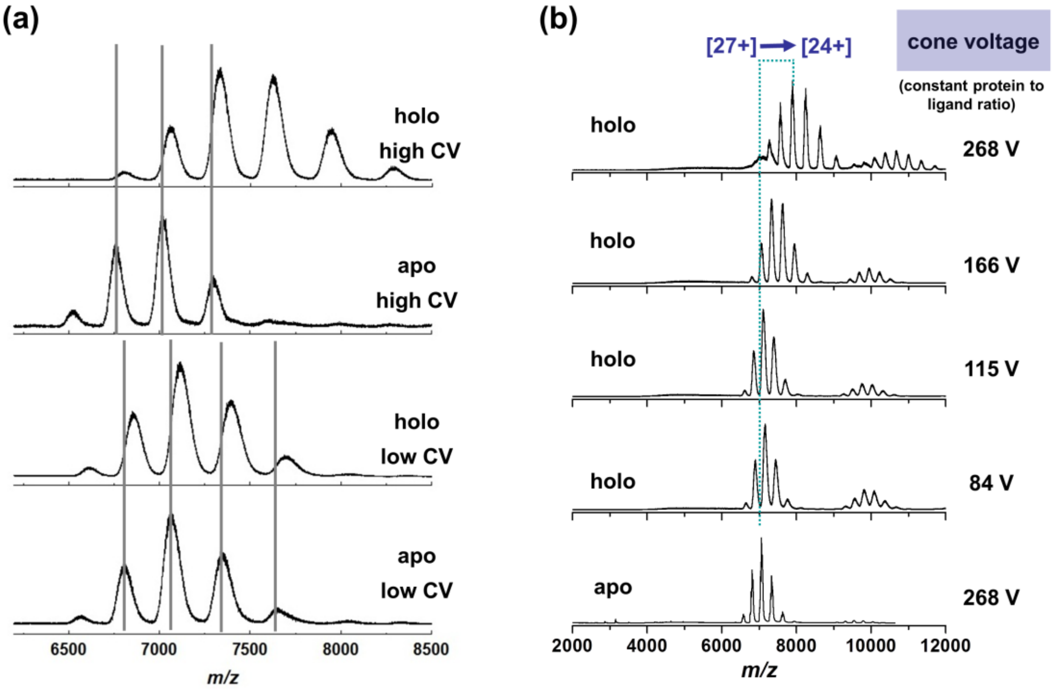


**Figure S3. Related to Figure 3 . (a) Mass spectra of MtATP-PRT in the presence and absence of 12 equivalents of L-histidine (denoted as 'holo'; 1 MtATP-PRT hexamer (H):12 ligand (L)) in 100 mM ammonium acetate at pH 6.8 at 115 V (low CV) and 166 V cone voltage (high CV), showing the mass shift associated with ligand binding. Moreover, a shift in the charge state envelope is noted at higher CV. Grey lines are used to help visualize the mass shift (based on apex of 'no ligand' peaks). (b) Dissociation of L-histidine upon increased cone voltage; 1 equivalent of MtATP-PRT incubated with 12 equivalents of L-histidine in 100 mM ammonium acetate pH 6.8 acquired at various cone voltages ranging from 84 – 268 V on Ultima API US Q-ToF mass spectrometer.**

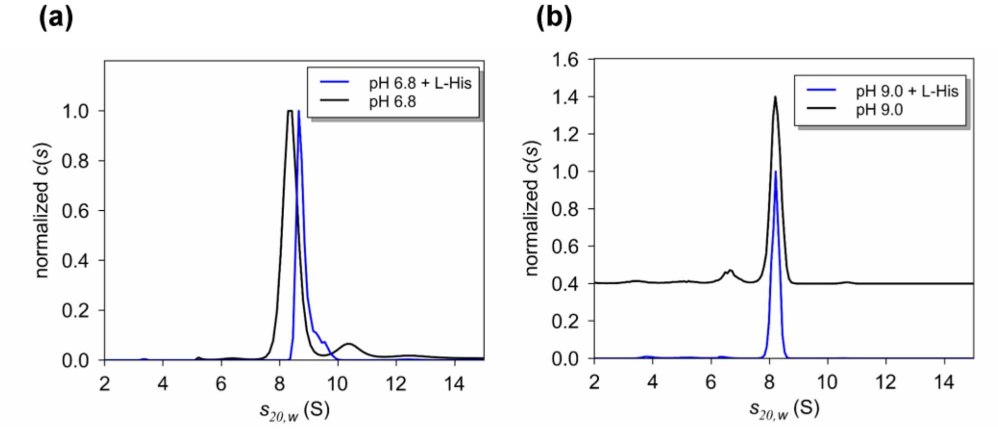


**Figure S4. Related to Figure 2b. Sedimentation coefficient distributions ($s_{20,w}$) of MtATP-PRT in 100 mM ammonium acetate at pH 6.8 (a) and pH 9 (b), in presence (blue trace) and in absence of L-histidine (black trace). More compact proteins experience less hydrodynamic friction, and thus have larger sedimentation coefficient.**

**Table S1. Related to Figure 2b. Results from the bead modelling of the apo and holo forms of MtATP-PRT compared to the data from AUC measurements: sedimentation coefficient (S), diffusion coefficient (D), hydrodynamic radius ($R_h$), radius of gyration ($R_g$) and total bead surface area ($A^b$).**

| | Experimental | | Bead model | |
|---|---|---|---|---|
| | **Apo** | **Holo** | **Apo** | **Holo** |
| **S** | 8.22 | 8.58 | 8.38 | 8.54 |
| **D ($cm/sec^2$)** | - | - | 4.50 | 4.54 |
| **$R_h$ (nm)** | 5.49 | 5.25 | 4.76 | 4.72 |
| **$R_g$ (nm)** | - | - | 3.75 | 3.75 |
| **$f/f_0$** | 1.37 | 1.28 | 1.27 | 1.26 |
| **$A^b$($nm^2$)** | - | - | 655 | 641 |

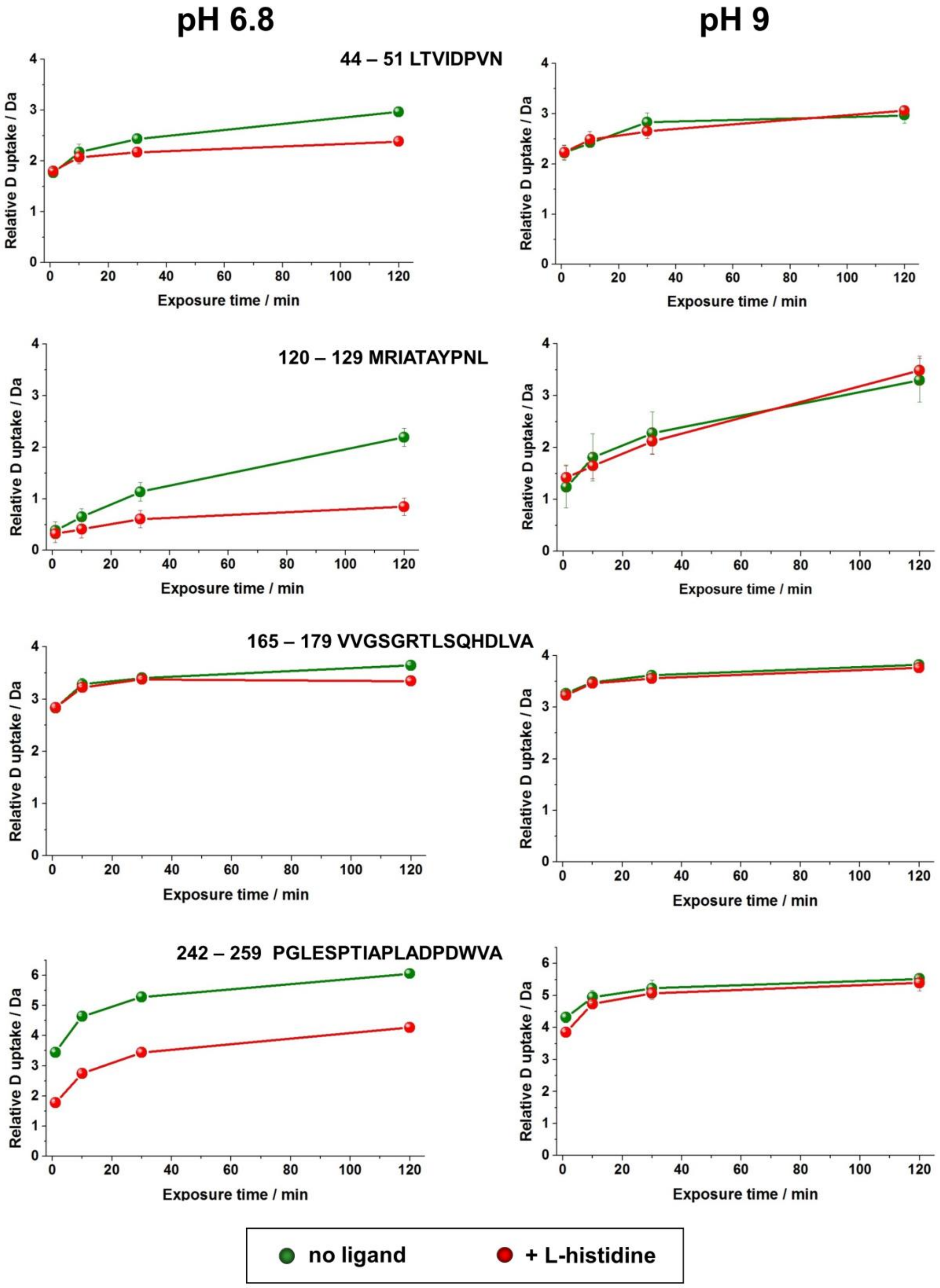


**Figure S5. Related to Figure 4. Example deuterium uptake curves of four chosen peptides of MtATP-PRT in presence (red) and in absence (green) of L-histidine at pH 6.8 (left) and pH 9 (right). Deuterium uptake was determined at four exposure time points: 1 minute, 10 minutes, 30 minutes and 120 minutes. D uptake data for all peptides identified is provided below.**

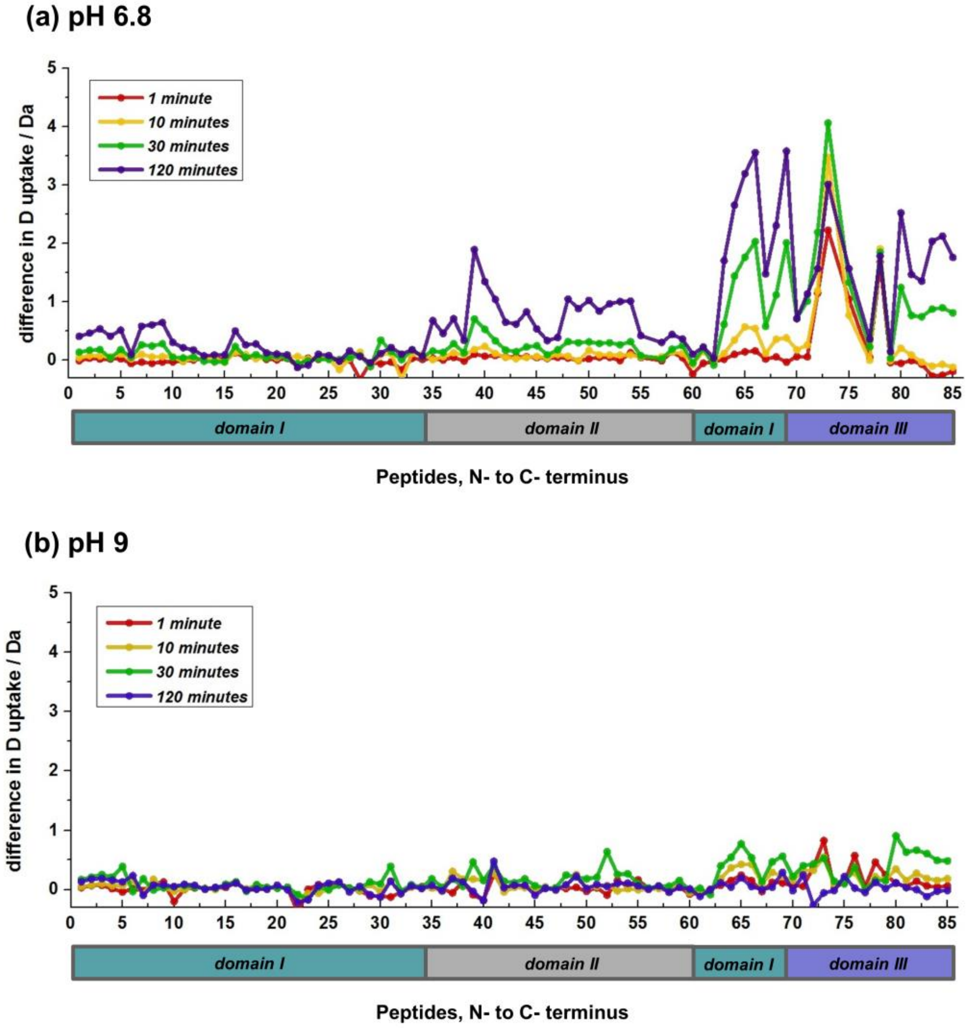


**Figure S6. Related to Figure 4. Difference in the absolute D uptake between L-histidine free and L-histidine bound MtATP-PRT at pH 6.8 (a) and pH 9 (b), measured at four deuterium exposure time points: 1 minute (red), 10 minutes (yellow), 30 minutes (green) and 120 minutes (blue); providing information about regions of ligand binding and conformational changes.**

**Table S2. Related to Figure 4. List of MtATP-PRT residues with altered D uptake due to L-histidine binding determined via HDX experiments; along with list of MtATP-PRT involved in binding with L-histidine, AMP and residues predicted to interact with ATP and PRPP.**

**HDX DATA**

| Residues | Change in D uptake | Peptide number |
|---|---|---|
| L44-F55 | long time scale: 120 min | 7-9 |
| L117-L129 | intermediate time scale: 30 min | 39-41 |
| V130-E151 | intermediate and long time scale: 30-120 min | 44-45 |
| I152-D164 | intermediate and long time scale: 30-120 min | 48-53 |
| V210-L234 | intermediate and long time scale: 30-120 min (significant) | 63-71 |
| D226-A259 | instant within 1 minute | 72, 73, 75, 78 |
| Y227-K235 | intermediate and long time scale: 30-120 min | 74 |
| V258-L285 | intermediate and long time scale: 30-120 min | 80-85 |

**X-RAY CRYSTALLOGRAPHY DATA (*6*)**

| Residues (HDX numbering) | Type of interaction |
|---|---|
| D228, L244, S246, T248, L263, A283 | L-histidine |
| D40, D43,D80, S100, Y126,D164-T171 | AMP |
| K19, K42, R59, R170 | ATP and PRPP (predicted) |

**Table S3a. Related to Figure 4. List of MtATP-PRT peptides identified after digestion with pepsin.**

| peptide # | Start residue | End residue | Maximum D uptake | Sequence |
|---|---|---|---|---|
| 1 | 12 | 27 | 13 | LRVAVPNKGALSEPAT |
| 2 | 12 | 28 | 14 | LRVAVPNKGALSEPATE |
| 3 | 12 | 30 | 16 | LRVAVPNKGALSEPATEIL |
| 4 | 13 | 28 | 13 | RVAVPNKGALSEPATE |
| 5 | 13 | 30 | 15 | RVAVPNKGALSEPATEIL |
| 6 | 29 | 35 | 6 | ILAEAGY |
| 7 | 44 | 51 | 6 | LTVIDPVN |
| 8 | 44 | 52 | 7 | LTVIDPVNN |
| 9 | 44 | 55 | 10 | LTVIDPVNNVEF |
| 10 | 45 | 55 | 9 | TVIDPVNNVEF |
| 11 | 46 | 55 | 8 | VIDPVNNVEF |
| 12 | 47 | 55 | 7 | IDPVNNVEF |
| 13 | 56 | 62 | 5 | FFLRPKD |
| 14 | 56 | 64 | 7 | FFLRPKDIA |
| 15 | 57 | 65 | 7 | FLRPKDIAI |
| 16 | 63 | 71 | 8 | IAIYVGSGE |
| 17 | 63 | 73 | 10 | IAIYVGSGELD |
| 18 | 63 | 74 | 11 | IAIYVGSGELDF |
| 19 | 65 | 74 | 9 | IYVGSGELDF |
| 20 | 66 | 73 | 7 | YVGSGELD |
| 21 | 66 | 74 | 8 | YVGSGELDF |
| 22 | 66 | 80 | 14 | YVGSGELDFGITGRD |
| 23 | 67 | 80 | 13 | VGSGELDFGITGRD |
| 24 | 72 | 80 | 8 | LDFGITGRD |
| 25 | 73 | 79 | 6 | DFGITGR |
| 26 | 73 | 80 | 7 | DFGITGRD |
| 27 | 81 | 88 | 7 | LVCDSGAQ |
| 28 | 81 | 91 | 10 | LVCDSGAQVRE |
| 29 | 84 | 93 | 9 | DSGAQVRERL |
| 30 | 89 | 102 | 13 | VRERLALGFGSSSF |
| 31 | 92 | 102 | 10 | RLALGFGSSSF |
| 32 | 94 | 101 | 7 | ALGFGSSS |
| 33 | 94 | 102 | 8 | ALGFGSSSF |
| 34 | 96 | 102 | 6 | GFGSSSF |
| 35 | 102 | 111 | 8 | FRYAAPAGRN |
| 36 | 103 | 111 | 7 | RYAAPAGRN |
| 37 | 103 | 117 | 13 | RYAAPAGRNWTTADL |
| 38 | 112 | 119 | 7 | WTTADLAG |
| 39 | 117 | 129 | 11 | LAGMRIATAYPNL |
| 40 | 120 | 129 | 8 | MRIATAYPNL |
| 41 | 121 | 129 | 7 | RIATAYPNL |
| 42 | 130 | 140 | 10 | VRKDLATKGIE |
| 43 | 130 | 141 | 11 | VRKDLATKGIEA |
| 44 | 130 | 142 | 12 | VRKDLATKGIEAT |
| 45 | 142 | 151 | 9 | TVIRLDGAVE |
| 46 | 143 | 149 | 6 | VIRLDGA |
| 47 | 143 | 151 | 8 | VIRLDGAVE |

| peptide # | Start residue | End residue | Maximum D uptake | Sequence |
|---|---|---|---|---|
| 48 | 152 | 159 | 7 | ISVQLGVA |
| 49 | 152 | 160 | 8 | ISVQLGVAD |
| 50 | 152 | 161 | 9 | ISVQLGVADA |
| 51 | 154 | 161 | 7 | VQLGVADA |
| 52 | 157 | 163 | 6 | GVADAIA |
| 53 | 157 | 164 | 7 | GVADAIAD |
| 54 | 160 | 179 | 19 | DAIADVVGSGRTLSQHDLVA |
| 55 | 162 | 179 | 17 | IADVVGSGRTLSQHDLVA |
| 56 | 164 | 179 | 15 | DVVGSGRTLSQHDLVA |
| 57 | 165 | 179 | 14 | VVGSGRTLSQHDLVA |
| 58 | 180 | 186 | 5 | FGEPLCD |
| 59 | 180 | 188 | 7 | FGEPLCDSE |
| 60 | 190 | 209 | 19 | VLIERAGTDGQDQTEARDQL |
| 61 | 191 | 199 | 8 | LIERAGTDG |
| 62 | 192 | 209 | 17 | IERAGTDGQDQTEARDQL |
| 63 | 210 | 218 | 8 | VARVQGVVF |
| 64 | 210 | 221 | 11 | VARVQGVVFGQQ |
| 65 | 210 | 222 | 12 | VARVQGVVFGQQY |
| 66 | 210 | 223 | 13 | VARVQGVVFGQQYL |
| 67 | 212 | 218 | 6 | RVQGVVF |
| 68 | 212 | 221 | 9 | RVQGVVFGQQ |
| 69 | 212 | 223 | 11 | RVQGVVFGQQYL |
| 70 | 225 | 233 | 7 | LDYDCPRSA |
| 71 | 225 | 234 | 8 | LDYDCPRSAL |
| 72 | 226 | 246 | 18 | DYDCPRSALKKATAITPGLES |
| 73 | 226 | 259 | 28 | DYDCPRSALKKATAITPGLESPTIAPLADPDWVA |
| 74 | 227 | 234 | 6 | YDCPRSAL |
| 75 | 235 | 246 | 10 | KKATAITPGLES |
| 76 | 235 | 259 | 14 | KKATAITPGLESPTIA |
| 77 | 237 | 245 | 7 | ATAITPGLE |
| 78 | 242 | 259 | 14 | PGLESPTIAPLADPDWVA |
| 79 | 249 | 257 | 6 | IAPLADPDW |
| 80 | 258 | 273 | 14 | VAIRALVPRRDVNGIM |
| 81 | 260 | 273 | 12 | IRALVPRRDVNGIM |
| 82 | 260 | 275 | 14 | IRALVPRRDVNGIMDE |
| 83 | 276 | 285 | 9 | LAAIGAKAIL |
| 84 | 277 | 285 | 8 | AAIGAKAIL |
| 85 | 279 | 285 | 6 | IGAKAIL |

**Table S3b. Related to Figure 4. Average relative D uptake at 1 minute, 10 minutes, 30 minutes and 120 minutes of exposure along with standard deviation values (SD) for apo MtATP-PRT at pH 6.8.**

| pH 6.8 | D UPTAKE /Da | | | | | | | |
|---|---|---|---|---|---|---|---|---|
| peptide # | 1 min | SD | 10 min | SD | 30 min | SD | 120 min | SD |
| 1 | 3.04 | 0.04 | 3.04 | 0.05 | 3.38 | 0.08 | 4.08 | 0.09 |
| 2 | 3.06 | 0.04 | 3.04 | 0.03 | 3.38 | 0.05 | 4.07 | 0.10 |
| 3 | 2.86 | 0.05 | 2.84 | 0.04 | 3.12 | 0.05 | 3.79 | 0.08 |
| 4 | 3.05 | 0.03 | 3.02 | 0.05 | 3.30 | 0.06 | 4.01 | 0.10 |
| 5 | 2.98 | 0.08 | 2.99 | 0.07 | 3.27 | 0.08 | 3.95 | 0.14 |
| 6 | 0.30 | 0.04 | 0.33 | 0.05 | 0.46 | 0.04 | 0.75 | 0.04 |
| 7 | 1.77 | 0.09 | 2.17 | 0.16 | 2.43 | 0.09 | 2.96 | 0.09 |
| 8 | 1.90 | 0.06 | 2.48 | 0.04 | 2.86 | 0.06 | 3.39 | 0.08 |
| 9 | 1.85 | 0.03 | 2.29 | 0.05 | 2.69 | 0.04 | 3.27 | 0.09 |
| 10 | 1.73 | 0.12 | 2.08 | 0.05 | 2.25 | 0.09 | 2.43 | 0.24 |
| 11 | 1.19 | 0.03 | 1.50 | 0.06 | 1.70 | 0.04 | 1.89 | 0.06 |
| 12 | 1.10 | 0.08 | 1.42 | 0.05 | 1.54 | 0.05 | 1.69 | 0.08 |
| 13 | 0.91 | 0.02 | 1.04 | 0.02 | 1.21 | 0.02 | 1.39 | 0.02 |
| 14 | 1.18 | 0.11 | 1.62 | 0.05 | 1.86 | 0.03 | 2.12 | 0.03 |
| 15 | 1.06 | 0.10 | 1.62 | 0.11 | 1.94 | 0.09 | 2.21 | 0.11 |
| 16 | 1.16 | 0.10 | 1.51 | 0.06 | 1.87 | 0.07 | 2.37 | 0.06 |
| 17 | 0.61 | 0.03 | 0.93 | 0.03 | 1.18 | 0.07 | 1.73 | 0.04 |
| 18 | 0.48 | 0.06 | 0.69 | 0.05 | 1.02 | 0.07 | 1.57 | 0.13 |
| 19 | 0.62 | 0.04 | 0.91 | 0.03 | 1.27 | 0.03 | 1.74 | 0.04 |
| 20 | 0.53 | 0.01 | 0.84 | 0.02 | 1.20 | 0.03 | 1.65 | 0.07 |
| 21 | 0.68 | 0.03 | 1.05 | 0.02 | 1.45 | 0.03 | 2.02 | 0.06 |
| 22 | 0.94 | 0.12 | 1.69 | 0.12 | 2.12 | 0.02 | 2.54 | 0.15 |
| 23 | 1.13 | 0.09 | 1.63 | 0.10 | 2.05 | 0.03 | 2.34 | 0.04 |
| 24 | 0.92 | 0.07 | 1.16 | 0.06 | 1.23 | 0.06 | 1.32 | 0.15 |
| 25 | 0.72 | 0.02 | 0.92 | 0.01 | 1.00 | 0.01 | 1.06 | 0.01 |
| 26 | 0.74 | 0.11 | 0.86 | 0.07 | 1.04 | 0.10 | 1.03 | 0.06 |
| 27 | 1.96 | 0.03 | 1.96 | 0.03 | 2.05 | 0.07 | 2.23 | 0.04 |
| 28 | 3.00 | 0.04 | 3.54 | 0.12 | 3.72 | 0.15 | 3.72 | 0.08 |
| 29 | 2.51 | 0.14 | 2.70 | 0.16 | 2.83 | 0.12 | 2.91 | 0.18 |
| 30 | 2.28 | 0.20 | 2.54 | 0.13 | 3.02 | 0.28 | 3.40 | 0.19 |
| 31 | 1.86 | 0.03 | 2.16 | 0.01 | 2.50 | 0.06 | 3.05 | 0.03 |
| 32 | 1.97 | 0.03 | 2.04 | 0.07 | 2.17 | 0.02 | 2.57 | 0.04 |
| 33 | 1.24 | 0.03 | 1.49 | 0.04 | 1.86 | 0.06 | 2.39 | 0.07 |
| 34 | 1.60 | 0.01 | 1.74 | 0.01 | 1.86 | 0.02 | 2.00 | 0.02 |
| 35 | 2.25 | 0.06 | 2.41 | 0.06 | 2.56 | 0.05 | 3.05 | 0.13 |
| 36 | 2.31 | 0.04 | 2.46 | 0.01 | 2.56 | 0.03 | 2.87 | 0.02 |
| 37 | 3.69 | 0.04 | 4.02 | 0.02 | 4.42 | 0.05 | 5.17 | 0.08 |
| 38 | 1.17 | 0.05 | 1.30 | 0.02 | 1.62 | 0.07 | 2.15 | 0.04 |
| 39 | 0.55 | 0.08 | 0.88 | 0.06 | 1.70 | 0.12 | 3.17 | 0.08 |
| 40 | 0.39 | 0.16 | 0.65 | 0.16 | 1.13 | 0.18 | 2.19 | 0.18 |
| 41 | 0.39 | 0.24 | 0.59 | 0.20 | 0.98 | 0.21 | 1.92 | 0.20 |
| 42 | 1.35 | 0.04 | 2.08 | 0.04 | 2.37 | 0.03 | 2.88 | 0.05 |
| 43 | 1.95 | 0.06 | 2.79 | 0.05 | 3.04 | 0.05 | 3.55 | 0.04 |
| 44 | 2.08 | 0.15 | 3.15 | 0.19 | 3.54 | 0.14 | 4.33 | 0.10 |
| 45 | 2.17 | 0.09 | 2.86 | 0.08 | 3.47 | 0.06 | 4.27 | 0.13 |

| peptide # | 1 min | SD | 10 min | SD | 30 min | SD | 120 min | SD |
|---|---|---|---|---|---|---|---|---|
| 46 | 1.33 | 0.05 | 1.56 | 0.06 | 1.80 | 0.08 | 2.17 | 0.07 |
| 47 | 2.01 | 0.04 | 2.63 | 0.06 | 3.12 | 0.04 | 3.75 | 0.02 |
| 48 | 1.37 | 0.03 | 1.75 | 0.03 | 2.07 | 0.01 | 2.91 | 0.03 |
| 49 | 1.45 | 0.11 | 1.83 | 0.07 | 2.19 | 0.06 | 2.97 | 0.08 |
| 50 | 1.70 | 0.17 | 2.16 | 0.36 | 2.43 | 0.04 | 3.29 | 0.10 |
| 51 | 0.70 | 0.03 | 1.00 | 0.03 | 1.27 | 0.05 | 1.86 | 0.05 |
| 52 | 0.40 | 0.06 | 0.38 | 0.04 | 0.61 | 0.07 | 1.29 | 0.07 |
| 53 | 0.45 | 0.08 | 0.44 | 0.02 | 0.68 | 0.08 | 1.48 | 0.12 |
| 54 | 6.01 | 0.07 | 6.14 | 0.07 | 6.72 | 0.12 | 7.97 | 0.11 |
| 55 | 4.44 | 0.11 | 4.84 | 0.09 | 5.23 | 0.10 | 6.00 | 0.07 |
| 57 | 2.83 | 0.04 | 3.28 | 0.12 | 3.40 | 0.07 | 3.64 | 0.03 |
| 58 | 0.71 | 0.03 | 0.70 | 0.05 | 0.82 | 0.09 | 1.28 | 0.04 |
| 59 | 0.56 | 0.04 | 0.66 | 0.04 | 0.96 | 0.05 | 1.46 | 0.07 |
| 60 | 6.35 | 0.18 | 6.93 | 0.10 | 7.17 | 0.16 | 7.62 | 0.15 |
| 61 | 1.16 | 0.09 | 1.48 | 0.12 | 1.87 | 0.15 | 2.40 | 0.08 |
| 62 | 5.97 | 0.06 | 6.40 | 0.04 | 6.60 | 0.13 | 7.01 | 0.12 |
| 63 | 0.24 | 0.02 | 0.32 | 0.02 | 0.86 | 0.02 | 2.02 | 0.02 |
| 64 | 0.58 | 0.05 | 0.95 | 0.07 | 2.31 | 0.06 | 3.89 | 0.06 |
| 65 | 0.63 | 0.11 | 1.19 | 0.08 | 2.72 | 0.03 | 4.56 | 0.07 |
| 66 | 0.61 | 0.04 | 1.21 | 0.05 | 2.93 | 0.03 | 4.82 | 0.15 |
| 67 | 0.15 | 0.03 | 0.22 | 0.02 | 0.69 | 0.04 | 1.66 | 0.03 |
| 68 | 0.57 | 0.06 | 0.99 | 0.07 | 1.94 | 0.07 | 3.52 | 0.14 |
| 69 | 0.49 | 0.07 | 1.03 | 0.07 | 2.83 | 0.16 | 4.75 | 0.08 |
| 70 | 0.72 | 0.05 | 1.08 | 0.10 | 2.02 | 0.07 | 2.63 | 0.08 |
| 71 | 0.82 | 0.03 | 1.26 | 0.04 | 2.44 | 0.03 | 3.22 | 0.03 |
| 72 | 4.31 | 0.31 | 5.62 | 0.18 | 7.56 | 0.30 | 8.65 | 0.27 |
| 73 | 6.10 | 0.18 | 9.13 | 0.17 | 11.32 | 0.26 | 13.03 | 0.31 |
| 74 | 0.80 | 0.09 | 1.17 | 0.10 | 2.05 | 0.09 | 2.57 | 0.09 |
| 75 | 2.55 | 0.04 | 3.07 | 0.17 | 4.11 | 0.02 | 4.83 | 0.17 |
| 77 | 1.48 | 0.06 | 1.75 | 0.02 | 2.35 | 0.02 | 2.91 | 0.05 |
| 78 | 3.45 | 0.09 | 4.65 | 0.06 | 5.29 | 0.17 | 6.06 | 0.15 |
| 79 | 1.20 | 0.03 | 1.82 | 0.14 | 2.07 | 0.14 | 2.38 | 0.07 |
| 80 | 1.49 | 0.08 | 1.81 | 0.09 | 3.28 | 0.07 | 5.24 | 0.16 |
| 81 | 1.53 | 0.03 | 1.77 | 0.04 | 2.84 | 0.03 | 4.24 | 0.07 |
| 82 | 1.68 | 0.13 | 2.00 | 0.17 | 3.29 | 0.18 | 4.65 | 0.18 |
| 83 | 0.53 | 0.02 | 1.50 | 0.03 | 2.86 | 0.02 | 4.32 | 0.03 |
| 84 | 0.50 | 0.02 | 1.38 | 0.03 | 2.61 | 0.03 | 3.92 | 0.04 |
| 85 | 0.42 | 0.04 | 1.08 | 0.06 | 2.23 | 0.10 | 3.26 | 0.16 |

**Table S3c. Related to Figure 4. Average relative D uptake at 1 minute, 10 minutes, 30 minutes and 120 minutes of exposure along with standard deviation values (SD) for MtATP-PRT in presence of L-histidine at pH 6.8.**

| pH 6.8 | D UPTAKE /Da | | | | | | | |
|---|---|---|---|---|---|---|---|---|
| peptide # | 1 min | SD | 10 min | SD | 30 min | SD | 120 min | SD |
| 1 | 3.05 | 0.03 | 3.00 | 0.03 | 3.25 | 0.03 | 3.67 | 0.06 |
| 2 | 3.03 | 0.04 | 2.97 | 0.04 | 3.22 | 0.02 | 3.60 | 0.05 |
| 3 | 2.84 | 0.03 | 2.77 | 0.02 | 2.94 | 0.03 | 3.25 | 0.06 |
| 4 | 3.03 | 0.04 | 2.97 | 0.03 | 3.26 | 0.03 | 3.60 | 0.06 |
| 5 | 2.95 | 0.08 | 2.89 | 0.09 | 3.09 | 0.08 | 3.43 | 0.10 |
| 6 | 0.36 | 0.06 | 0.31 | 0.06 | 0.41 | 0.03 | 0.65 | 0.04 |
| 7 | 1.80 | 0.05 | 2.07 | 0.13 | 2.17 | 0.04 | 2.38 | 0.04 |
| 8 | 1.95 | 0.05 | 2.43 | 0.04 | 2.61 | 0.04 | 2.78 | 0.04 |
| 9 | 1.89 | 0.11 | 2.23 | 0.05 | 2.41 | 0.10 | 2.62 | 0.06 |
| 10 | 1.76 | 0.08 | 2.02 | 0.17 | 2.20 | 0.05 | 2.13 | 0.12 |
| 11 | 1.21 | 0.02 | 1.53 | 0.04 | 1.66 | 0.05 | 1.67 | 0.02 |
| 12 | 1.10 | 0.06 | 1.39 | 0.06 | 1.48 | 0.08 | 1.51 | 0.06 |
| 13 | 0.87 | 0.02 | 1.03 | 0.02 | 1.23 | 0.02 | 1.32 | 0.03 |
| 14 | 1.12 | 0.05 | 1.58 | 0.05 | 1.89 | 0.05 | 2.03 | 0.05 |
| 15 | 1.06 | 0.08 | 1.66 | 0.14 | 1.96 | 0.06 | 2.12 | 0.09 |
| 16 | 1.04 | 0.03 | 1.37 | 0.02 | 1.64 | 0.04 | 1.87 | 0.05 |
| 17 | 0.56 | 0.04 | 0.83 | 0.06 | 1.13 | 0.03 | 1.47 | 0.02 |
| 18 | 0.42 | 0.07 | 0.67 | 0.09 | 0.93 | 0.07 | 1.29 | 0.06 |
| 19 | 0.61 | 0.03 | 0.86 | 0.03 | 1.23 | 0.03 | 1.62 | 0.03 |
| 20 | 0.53 | 0.02 | 0.80 | 0.03 | 1.13 | 0.01 | 1.54 | 0.03 |
| 21 | 0.65 | 0.03 | 1.00 | 0.02 | 1.42 | 0.02 | 1.93 | 0.04 |
| 22 | 1.05 | 0.05 | 1.63 | 0.04 | 2.18 | 0.11 | 2.67 | 0.05 |
| 23 | 1.10 | 0.11 | 1.61 | 0.10 | 2.06 | 0.09 | 2.42 | 0.05 |
| 24 | 0.90 | 0.05 | 1.11 | 0.06 | 1.21 | 0.06 | 1.21 | 0.07 |
| 25 | 0.69 | 0.02 | 0.89 | 0.01 | 0.98 | 0.01 | 0.98 | 0.02 |
| 26 | 0.76 | 0.11 | 1.02 | 0.11 | 1.03 | 0.11 | 1.05 | 0.12 |
| 27 | 1.96 | 0.02 | 1.93 | 0.03 | 2.01 | 0.04 | 2.07 | 0.04 |
| 28 | 3.33 | 0.16 | 3.40 | 0.05 | 3.64 | 0.01 | 3.66 | 0.08 |
| 29 | 2.56 | 0.18 | 2.80 | 0.10 | 2.94 | 0.08 | 2.96 | 0.15 |
| 30 | 2.35 | 0.07 | 2.45 | 0.15 | 2.68 | 0.13 | 3.28 | 0.14 |
| 31 | 1.89 | 0.05 | 2.04 | 0.02 | 2.35 | 0.05 | 2.83 | 0.06 |
| 32 | 2.14 | 0.14 | 2.38 | 0.06 | 2.17 | 0.07 | 2.47 | 0.03 |
| 33 | 1.20 | 0.04 | 1.37 | 0.03 | 1.68 | 0.04 | 2.21 | 0.06 |
| 34 | 1.59 | 0.04 | 1.64 | 0.03 | 1.80 | 0.02 | 1.93 | 0.06 |
| 35 | 2.21 | 0.06 | 2.40 | 0.07 | 2.40 | 0.03 | 2.37 | 0.05 |
| 36 | 2.31 | 0.04 | 2.40 | 0.03 | 2.43 | 0.04 | 2.41 | 0.04 |
| 37 | 3.64 | 0.04 | 3.89 | 0.02 | 4.14 | 0.02 | 4.46 | 0.08 |
| 38 | 1.18 | 0.05 | 1.21 | 0.04 | 1.49 | 0.03 | 1.81 | 0.04 |
| 39 | 0.45 | 0.04 | 0.69 | 0.05 | 0.99 | 0.04 | 1.28 | 0.04 |
| 40 | 0.32 | 0.17 | 0.41 | 0.17 | 0.61 | 0.17 | 0.85 | 0.17 |
| 41 | 0.31 | 0.28 | 0.48 | 0.27 | 0.64 | 0.27 | 0.88 | 0.28 |
| 42 | 1.30 | 0.03 | 2.03 | 0.03 | 2.20 | 0.03 | 2.23 | 0.04 |
| 43 | 1.90 | 0.04 | 2.76 | 0.04 | 2.89 | 0.05 | 2.93 | 0.05 |
| 44 | 2.02 | 0.10 | 3.10 | 0.15 | 3.32 | 0.18 | 3.51 | 0.22 |
| 45 | 2.13 | 0.08 | 2.80 | 0.07 | 3.23 | 0.06 | 3.73 | 0.07 |

| peptide # | 1 min | SD | 10 min | SD | 30 min | SD | 120 min | SD |
|---|---|---|---|---|---|---|---|---|
| 46 | 1.30 | 0.03 | 1.52 | 0.06 | 1.71 | 0.09 | 1.83 | 0.07 |
| 47 | 1.96 | 0.02 | 2.53 | 0.02 | 2.94 | 0.03 | 3.36 | 0.03 |
| 48 | 1.33 | 0.01 | 1.69 | 0.01 | 1.75 | 0.02 | 1.86 | 0.03 |
| 49 | 1.46 | 0.05 | 1.84 | 0.06 | 1.90 | 0.03 | 2.09 | 0.06 |
| 50 | 1.68 | 0.31 | 1.99 | 0.03 | 2.11 | 0.04 | 2.27 | 0.06 |
| 51 | 0.65 | 0.03 | 0.91 | 0.01 | 0.98 | 0.01 | 1.02 | 0.01 |
| 52 | 0.35 | 0.03 | 0.29 | 0.03 | 0.31 | 0.03 | 0.33 | 0.04 |
| 53 | 0.45 | 0.02 | 0.36 | 0.02 | 0.42 | 0.07 | 0.48 | 0.05 |
| 54 | 5.88 | 0.09 | 6.05 | 0.04 | 6.40 | 0.05 | 6.96 | 0.12 |
| 55 | 4.39 | 0.11 | 4.78 | 0.08 | 5.15 | 0.07 | 5.58 | 0.10 |
| 57 | 2.84 | 0.03 | 3.23 | 0.03 | 3.38 | 0.03 | 3.34 | 0.05 |
| 58 | 0.57 | 0.05 | 0.57 | 0.04 | 0.64 | 0.04 | 0.84 | 0.05 |
| 59 | 0.52 | 0.02 | 0.55 | 0.03 | 0.71 | 0.04 | 1.09 | 0.04 |
| 60 | 6.58 | 0.16 | 7.01 | 0.16 | 7.22 | 0.11 | 7.52 | 0.13 |
| 61 | 1.21 | 0.06 | 1.32 | 0.10 | 1.67 | 0.11 | 2.17 | 0.07 |
| 62 | 5.99 | 0.03 | 6.42 | 0.03 | 6.69 | 0.03 | 6.98 | 0.10 |
| 63 | 0.22 | 0.01 | 0.21 | 0.01 | 0.24 | 0.01 | 0.31 | 0.01 |
| 64 | 0.49 | 0.03 | 0.60 | 0.03 | 0.87 | 0.02 | 1.24 | 0.03 |
| 65 | 0.49 | 0.04 | 0.62 | 0.05 | 0.96 | 0.02 | 1.37 | 0.04 |
| 66 | 0.45 | 0.05 | 0.67 | 0.04 | 0.90 | 0.10 | 1.26 | 0.09 |
| 67 | 0.13 | 0.03 | 0.11 | 0.03 | 0.11 | 0.02 | 0.18 | 0.02 |
| 68 | 0.51 | 0.08 | 0.63 | 0.07 | 0.82 | 0.07 | 1.22 | 0.06 |
| 69 | 0.52 | 0.11 | 0.65 | 0.07 | 0.82 | 0.11 | 1.16 | 0.07 |
| 70 | 0.66 | 0.05 | 0.90 | 0.03 | 1.30 | 0.03 | 1.92 | 0.06 |
| 71 | 0.76 | 0.03 | 1.00 | 0.03 | 1.43 | 0.03 | 2.08 | 0.05 |
| 72 | 3.16 | 0.13 | 4.43 | 0.34 | 5.37 | 0.10 | 7.08 | 0.17 |
| 73 | 3.87 | 0.18 | 5.65 | 0.17 | 7.26 | 0.21 | 10.01 | 0.20 |
| 74 | 0.81 | 0.08 | 1.02 | 0.07 | 1.41 | 0.07 | 2.02 | 0.09 |
| 75 | 1.50 | 0.07 | 2.30 | 0.07 | 2.78 | 0.06 | 3.26 | 0.26 |
| 77 | 1.43 | 0.06 | 1.76 | 0.02 | 2.12 | 0.06 | 2.55 | 0.08 |
| 78 | 1.77 | 0.04 | 2.74 | 0.08 | 3.44 | 0.13 | 4.28 | 0.11 |
| 79 | 1.24 | 0.05 | 1.83 | 0.05 | 2.03 | 0.06 | 2.24 | 0.06 |
| 80 | 1.54 | 0.14 | 1.60 | 0.15 | 2.04 | 0.10 | 2.72 | 0.15 |
| 81 | 1.54 | 0.02 | 1.69 | 0.03 | 2.07 | 0.03 | 2.77 | 0.05 |
| 82 | 1.75 | 0.13 | 2.03 | 0.12 | 2.55 | 0.13 | 3.30 | 0.14 |
| 83 | 0.80 | 0.01 | 1.61 | 0.01 | 1.99 | 0.03 | 2.29 | 0.03 |
| 84 | 0.76 | 0.01 | 1.45 | 0.01 | 1.72 | 0.01 | 1.80 | 0.03 |
| 85 | 0.61 | 0.04 | 1.20 | 0.05 | 1.42 | 0.07 | 1.50 | 0.06 |

**Table S3d. Related to Figure 4. Average relative D uptake at 1 minute, 10 minutes, 30 minutes and 120 minutes of exposure along with standard deviation values (SD) for apo MtATP-PRT at pH 9.**

| pH 9 | D UPTAKE /Da | | | | | | | |
|---|---|---|---|---|---|---|---|---|
| peptide # | 1 min | SD | 10 min | SD | 30 min | SD | 120 min | SD |
| 1 | 4.13 | 0.13 | 4.31 | 0.13 | 4.88 | 0.13 | 5.56 | 0.39 |
| 2 | 4.30 | 0.04 | 4.40 | 0.09 | 5.03 | 0.11 | 5.74 | 0.13 |
| 3 | 4.40 | 0.02 | 4.60 | 0.05 | 5.24 | 0.06 | 6.05 | 0.14 |
| 4 | 4.01 | 0.15 | 4.25 | 0.12 | 4.81 | 0.18 | 5.44 | 0.22 |
| 5 | 4.71 | 2.24 | 4.82 | 1.85 | 5.39 | 1.55 | 6.08 | 1.05 |
| 6 | 0.82 | 0.13 | 0.96 | 0.05 | 1.09 | 0.17 | 1.59 | 0.09 |
| 7 | 2.21 | 0.15 | 2.42 | 0.08 | 2.83 | 0.19 | 2.97 | 0.16 |
| 8 | 2.43 | 0.11 | 2.75 | 0.12 | 3.19 | 0.11 | 3.47 | 0.12 |
| 9 | 3.79 | 0.27 | 4.01 | 0.25 | 4.27 | 0.23 | 4.53 | 0.22 |
| 10 | 2.24 | 1.09 | 2.45 | 0.81 | 2.82 | 1.35 | 3.01 | 1.51 |
| 11 | 1.55 | 0.04 | 1.85 | 0.05 | 2.06 | 0.06 | 2.34 | 0.06 |
| 12 | 1.44 | 0.08 | 1.72 | 0.06 | 1.86 | 0.08 | 2.12 | 0.09 |
| 13 | 1.06 | 0.02 | 1.24 | 0.02 | 1.39 | 0.02 | 1.54 | 0.03 |
| 14 | 1.49 | 0.03 | 1.89 | 0.03 | 2.14 | 0.03 | 2.41 | 0.04 |
| 15 | 1.51 | 0.08 | 1.99 | 0.08 | 2.27 | 0.07 | 2.56 | 0.08 |
| 16 | 1.60 | 0.26 | 1.98 | 0.44 | 2.37 | 0.38 | 2.68 | 0.23 |
| 17 | 0.89 | 0.17 | 1.26 | 0.15 | 1.59 | 0.25 | 2.13 | 0.45 |
| 18 | 0.91 | 0.12 | 1.31 | 0.08 | 1.68 | 0.15 | 2.38 | 0.15 |
| 19 | 1.12 | 0.04 | 1.49 | 0.04 | 1.94 | 0.04 | 2.36 | 0.06 |
| 20 | 0.89 | 0.02 | 1.25 | 0.03 | 1.61 | 0.01 | 2.03 | 0.02 |
| 21 | 1.20 | 0.03 | 1.64 | 0.02 | 2.12 | 0.04 | 2.53 | 0.02 |
| 22 | 1.69 | 0.24 | 2.67 | 0.17 | 3.42 | 0.23 | 3.82 | 0.24 |
| 23 | 1.75 | 0.27 | 2.35 | 0.24 | 2.87 | 0.21 | 3.33 | 0.30 |
| 24 | 1.38 | 0.03 | 1.49 | 0.14 | 1.66 | 0.16 | 1.92 | 0.17 |
| 25 | 0.97 | 0.03 | 1.13 | 0.03 | 1.23 | 0.06 | 1.42 | 0.08 |
| 26 | 1.05 | 0.03 | 1.20 | 0.12 | 1.31 | 0.07 | 1.54 | 0.11 |
| 27 | 2.20 | 0.04 | 2.10 | 0.06 | 2.17 | 0.09 | 2.27 | 0.16 |
| 28 | 3.68 | 0.22 | 3.80 | 0.12 | 3.96 | 0.13 | 4.07 | 0.34 |
| 29 | 2.90 | 0.19 | 3.18 | 0.22 | 3.40 | 0.22 | 3.46 | 0.27 |
| 30 | 2.62 | 0.18 | 2.92 | 0.08 | 3.37 | 0.12 | 3.99 | 0.27 |
| 31 | 2.26 | 0.05 | 2.60 | 0.60 | 3.16 | 0.76 | 3.55 | 0.67 |
| 32 | 2.30 | 0.09 | 2.24 | 0.02 | 2.36 | 0.03 | 2.65 | 0.07 |
| 33 | 1.53 | 0.04 | 1.74 | 0.04 | 2.04 | 0.05 | 2.48 | 0.07 |
| 34 | 1.64 | 0.02 | 1.68 | 0.03 | 1.77 | 0.07 | 1.88 | 0.04 |
| 35 | 2.80 | 0.07 | 2.86 | 0.06 | 3.11 | 0.10 | 3.54 | 0.11 |
| 36 | 2.76 | 0.06 | 2.80 | 0.07 | 2.94 | 0.07 | 3.16 | 0.09 |
| 37 | 4.80 | 0.25 | 4.86 | 1.18 | 5.48 | 1.48 | 6.04 | 1.69 |
| 38 | 1.70 | 0.44 | 1.97 | 0.75 | 2.20 | 0.58 | 2.59 | 0.63 |
| 39 | 1.72 | 0.23 | 2.32 | 0.26 | 3.08 | 0.27 | 4.05 | 0.21 |
| 40 | 1.24 | 0.40 | 1.81 | 0.45 | 2.28 | 0.41 | 3.30 | 0.42 |
| 41 | 1.36 | 0.58 | 1.60 | 0.26 | 2.16 | 0.72 | 3.19 | 0.36 |
| 42 | 2.18 | 0.04 | 2.59 | 0.05 | 2.91 | 0.03 | 3.34 | 0.07 |
| 43 | 2.81 | 0.05 | 3.19 | 0.03 | 3.46 | 0.02 | 3.93 | 0.06 |
| 44 | 3.23 | 0.15 | 3.73 | 0.14 | 4.17 | 0.16 | 4.86 | 0.12 |
| 45 | 2.69 | 0.10 | 3.18 | 0.06 | 3.78 | 0.07 | 4.32 | 0.14 |

| peptide # | 1 min | SD | 10 min | SD | 30 min | SD | 120 min | SD |
|---|---|---|---|---|---|---|---|---|
| 46 | 1.60 | 0.09 | 1.72 | 0.06 | 1.89 | 0.06 | 2.17 | 0.07 |
| 47 | 2.45 | 0.03 | 2.82 | 0.02 | 3.32 | 0.02 | 3.70 | 0.06 |
| 48 | 1.90 | 0.18 | 2.26 | 0.13 | 2.68 | 0.13 | 3.47 | 0.16 |
| 49 | 2.00 | 0.51 | 2.55 | 0.50 | 3.01 | 0.46 | 3.69 | 0.49 |
| 50 | 2.31 | 0.14 | 2.65 | 0.13 | 3.17 | 0.22 | 3.95 | 0.15 |
| 51 | 1.12 | 0.06 | 1.38 | 0.03 | 1.78 | 0.11 | 2.31 | 0.05 |
| 52 | 0.62 | 0.11 | 0.83 | 0.06 | 1.61 | 0.36 | 1.71 | 0.08 |
| 53 | 1.10 | 0.13 | 1.09 | 0.10 | 1.52 | 0.08 | 2.11 | 0.10 |
| 54 | 6.82 | 0.07 | 7.00 | 0.11 | 7.72 | 0.10 | 8.69 | 0.19 |
| 55 | 5.00 | 0.03 | 5.21 | 0.07 | 5.67 | 0.07 | 6.22 | 0.15 |
| 56 | 5.05 | 0.03 | 5.22 | 0.05 | 5.47 | 0.07 | 5.83 | 0.14 |
| 57 | 3.27 | 0.03 | 3.48 | 0.04 | 3.62 | 0.04 | 3.82 | 0.10 |
| 58 | 0.67 | 0.04 | 0.77 | 0.05 | 0.94 | 0.04 | 1.23 | 0.06 |
| 59 | 0.73 | 0.10 | 0.91 | 0.06 | 1.19 | 0.06 | 1.66 | 0.09 |
| 60 | 7.13 | 0.13 | 7.29 | 0.08 | 7.51 | 0.13 | 7.98 | 0.16 |
| 61 | 1.33 | 0.07 | 1.66 | 0.11 | 1.94 | 0.13 | 2.32 | 0.06 |
| 62 | 6.65 | 0.05 | 6.70 | 0.07 | 6.84 | 0.10 | 7.15 | 0.15 |
| 63 | 0.97 | 0.05 | 1.31 | 0.05 | 2.10 | 0.08 | 3.02 | 0.17 |
| 64 | 1.84 | 0.07 | 2.56 | 0.08 | 3.85 | 0.07 | 4.84 | 0.10 |
| 65 | 2.09 | 0.59 | 2.85 | 0.50 | 4.56 | 0.31 | 5.62 | 0.34 |
| 66 | 2.19 | 0.47 | 3.07 | 0.52 | 4.68 | 0.27 | 5.81 | 0.26 |
| 67 | 1.13 | 0.19 | 1.34 | 0.21 | 1.67 | 0.19 | 2.28 | 0.07 |
| 68 | 1.40 | 0.11 | 1.99 | 0.19 | 3.24 | 0.08 | 4.19 | 0.06 |
| 69 | 1.87 | 0.39 | 2.63 | 0.48 | 4.22 | 0.33 | 5.55 | 0.32 |
| 70 | 1.09 | 0.03 | 1.61 | 0.04 | 2.41 | 0.04 | 2.64 | 0.07 |
| 71 | 1.57 | 0.73 | 2.39 | 0.71 | 3.25 | 0.60 | 3.86 | 0.81 |
| 72 | 5.51 | 0.14 | 6.81 | 0.11 | 8.42 | 0.27 | 8.53 | 0.37 |
| 73 | 8.21 | 0.23 | 10.36 | 0.22 | 12.37 | 0.18 | 13.18 | 0.38 |
| 74 | 1.01 | 0.11 | 1.49 | 0.12 | 2.21 | 0.11 | 2.48 | 0.13 |
| 75 | 3.72 | 0.46 | 4.17 | 0.48 | 4.68 | 0.60 | 4.82 | 0.70 |
| 76 | 5.58 | 0.05 | 6.71 | 0.04 | 7.77 | 0.09 | 8.31 | 0.17 |
| 77 | 1.77 | 0.11 | 2.12 | 0.08 | 2.53 | 0.13 | 2.86 | 0.11 |
| 78 | 4.32 | 0.16 | 4.95 | 0.20 | 5.22 | 0.26 | 5.52 | 0.08 |
| 79 | 1.81 | 0.63 | 2.19 | 0.59 | 2.23 | 0.47 | 2.48 | 0.56 |
| 80 | 1.74 | 0.08 | 2.41 | 0.20 | 4.29 | 0.14 | 5.67 | 0.20 |
| 81 | 1.76 | 0.04 | 2.18 | 0.06 | 3.60 | 0.11 | 4.57 | 0.12 |
| 82 | 1.94 | 0.13 | 2.53 | 0.18 | 4.03 | 0.19 | 5.10 | 0.32 |
| 83 | 1.11 | 0.06 | 2.13 | 0.08 | 3.75 | 0.09 | 4.67 | 0.11 |
| 84 | 1.02 | 0.05 | 1.94 | 0.07 | 3.31 | 0.12 | 4.29 | 0.09 |
| 85 | 0.88 | 0.06 | 1.65 | 0.10 | 2.85 | 0.14 | 3.65 | 0.12 |

**Table S3e. Related to Figure 4. Average relative D uptake at 1 minute, 10 minutes, 30 minutes and 120 minutes of exposure along with standard deviation values (SD) for MtATP-PRT in presence of L-histidine at pH 9.**

| pH 9 | D UPTAKE / Da | | | | | | | |
|---|---|---|---|---|---|---|---|---|
| peptide # | 1 min | SD | 10 min | SD | 30 min | SD | 120 min | SD |
| 1 | 4.09 | 0.03 | 4.26 | 0.04 | 4.71 | 0.05 | 5.42 | 0.28 |
| 2 | 4.23 | 0.02 | 4.33 | 0.06 | 4.82 | 0.18 | 5.57 | 0.05 |
| 3 | 4.33 | 0.04 | 4.50 | 0.06 | 4.98 | 0.07 | 5.87 | 0.07 |
| 4 | 4.00 | 0.13 | 4.18 | 0.08 | 4.60 | 0.20 | 5.28 | 0.20 |
| 5 | 4.75 | 2.40 | 4.75 | 1.90 | 5.00 | 0.75 | 5.95 | 1.58 |
| 6 | 0.80 | 0.06 | 0.80 | 0.14 | 1.13 | 0.05 | 1.36 | 0.07 |
| 7 | 2.23 | 0.14 | 2.49 | 0.16 | 2.64 | 0.14 | 3.06 | 0.09 |
| 8 | 2.33 | 0.05 | 2.58 | 0.13 | 3.20 | 0.16 | 3.39 | 0.09 |
| 9 | 3.67 | 0.24 | 3.98 | 0.25 | 4.24 | 0.24 | 4.45 | 0.18 |
| 10 | 2.45 | 1.11 | 2.48 | 0.84 | 2.78 | 1.32 | 2.96 | 1.06 |
| 11 | 1.55 | 0.04 | 1.85 | 0.04 | 1.99 | 0.06 | 2.25 | 0.05 |
| 12 | 1.41 | 0.06 | 1.67 | 0.07 | 1.82 | 0.07 | 2.05 | 0.07 |
| 13 | 1.04 | 0.01 | 1.22 | 0.02 | 1.37 | 0.02 | 1.54 | 0.02 |
| 14 | 1.46 | 0.02 | 1.89 | 0.04 | 2.12 | 0.02 | 2.37 | 0.02 |
| 15 | 1.47 | 0.04 | 1.91 | 0.11 | 2.18 | 0.07 | 2.50 | 0.04 |
| 16 | 1.49 | 0.23 | 1.85 | 0.10 | 2.23 | 0.18 | 2.57 | 0.13 |
| 17 | 0.90 | 0.10 | 1.24 | 0.13 | 1.62 | 0.14 | 2.14 | 0.24 |
| 18 | 0.90 | 0.08 | 1.24 | 0.07 | 1.60 | 0.07 | 2.37 | 0.23 |
| 19 | 1.09 | 0.02 | 1.45 | 0.03 | 1.90 | 0.03 | 2.35 | 0.02 |
| 20 | 0.85 | 0.02 | 1.22 | 0.03 | 1.58 | 0.03 | 1.95 | 0.02 |
| 21 | 1.17 | 0.03 | 1.61 | 0.02 | 2.08 | 0.03 | 2.54 | 0.02 |
| 22 | 2.08 | 0.24 | 2.80 | 0.12 | 3.50 | 0.16 | 4.04 | 0.12 |
| 23 | 1.75 | 0.27 | 2.42 | 0.24 | 3.03 | 0.26 | 3.50 | 0.18 |
| 24 | 1.29 | 0.07 | 1.55 | 0.09 | 1.62 | 0.15 | 1.85 | 0.13 |
| 25 | 0.94 | 0.03 | 1.13 | 0.04 | 1.23 | 0.07 | 1.31 | 0.05 |
| 26 | 0.94 | 0.06 | 1.12 | 0.05 | 1.23 | 0.08 | 1.41 | 0.10 |
| 27 | 2.17 | 0.02 | 2.09 | 0.02 | 2.15 | 0.03 | 2.31 | 0.06 |
| 28 | 3.70 | 0.11 | 3.82 | 0.13 | 3.93 | 0.15 | 4.02 | 0.27 |
| 29 | 3.01 | 0.27 | 3.09 | 0.29 | 3.27 | 0.23 | 3.55 | 0.14 |
| 30 | 2.73 | 0.10 | 2.96 | 0.16 | 3.28 | 0.11 | 4.12 | 0.10 |
| 31 | 2.38 | 0.60 | 2.46 | 0.04 | 2.77 | 0.04 | 3.41 | 0.53 |
| 32 | 2.34 | 0.15 | 2.31 | 0.10 | 2.38 | 0.02 | 2.72 | 0.05 |
| 33 | 1.49 | 0.03 | 1.70 | 0.03 | 1.96 | 0.04 | 2.42 | 0.06 |
| 34 | 1.60 | 0.02 | 1.66 | 0.02 | 1.73 | 0.03 | 1.86 | 0.01 |
| 35 | 2.76 | 0.11 | 2.84 | 0.06 | 2.93 | 0.11 | 3.47 | 0.10 |
| 36 | 2.74 | 0.05 | 2.83 | 0.08 | 2.90 | 0.09 | 3.18 | 0.08 |
| 37 | 4.86 | 1.31 | 4.55 | 0.12 | 5.30 | 1.86 | 5.85 | 0.79 |
| 38 | 1.57 | 0.51 | 1.79 | 0.68 | 2.15 | 0.65 | 2.46 | 0.69 |
| 39 | 1.81 | 0.24 | 2.14 | 0.27 | 2.62 | 0.21 | 4.08 | 0.16 |
| 40 | 1.42 | 0.25 | 1.65 | 0.25 | 2.12 | 0.24 | 3.48 | 0.28 |
| 41 | 1.11 | 0.53 | 1.33 | 0.58 | 1.76 | 0.68 | 2.72 | 0.12 |
| 42 | 2.14 | 0.04 | 2.63 | 0.13 | 2.77 | 0.03 | 3.31 | 0.04 |
| 43 | 2.75 | 0.04 | 3.15 | 0.02 | 3.35 | 0.04 | 3.86 | 0.07 |
| 44 | 3.17 | 0.13 | 3.70 | 0.13 | 3.99 | 0.12 | 4.79 | 0.14 |
| 45 | 2.69 | 0.10 | 3.17 | 0.07 | 3.71 | 0.08 | 4.41 | 0.10 |

| peptide # | 1 min | SD | 10 min | SD | 30 min | SD | 120 min | SD |
|---|---|---|---|---|---|---|---|---|
| 46 | 1.58 | 0.09 | 1.67 | 0.07 | 1.87 | 0.09 | 2.15 | 0.05 |
| 47 | 2.43 | 0.01 | 2.83 | 0.02 | 3.29 | 0.06 | 3.70 | 0.02 |
| 48 | 1.88 | 0.12 | 2.19 | 0.13 | 2.43 | 0.15 | 3.39 | 0.11 |
| 49 | 1.96 | 0.68 | 2.29 | 0.67 | 2.80 | 0.71 | 3.46 | 0.63 |
| 50 | 2.34 | 0.19 | 2.62 | 0.18 | 2.99 | 0.22 | 3.94 | 0.19 |
| 51 | 1.09 | 0.06 | 1.33 | 0.03 | 1.58 | 0.10 | 2.23 | 0.15 |
| 52 | 0.71 | 0.04 | 0.76 | 0.04 | 0.97 | 0.09 | 1.65 | 0.05 |
| 53 | 0.96 | 0.10 | 1.12 | 0.05 | 1.26 | 0.08 | 2.00 | 0.14 |
| 54 | 6.77 | 0.05 | 6.99 | 0.07 | 7.46 | 0.07 | 8.58 | 0.17 |
| 55 | 4.83 | 0.10 | 5.22 | 0.06 | 5.57 | 0.06 | 6.16 | 0.07 |
| 56 | 5.03 | 0.02 | 5.22 | 0.04 | 5.45 | 0.04 | 5.82 | 0.07 |
| 57 | 3.23 | 0.05 | 3.46 | 0.03 | 3.56 | 0.05 | 3.76 | 0.04 |
| 58 | 0.71 | 0.03 | 0.81 | 0.11 | 0.88 | 0.19 | 1.28 | 0.09 |
| 59 | 0.69 | 0.08 | 0.79 | 0.06 | 1.03 | 0.08 | 1.62 | 0.08 |
| 60 | 7.20 | 0.15 | 7.35 | 0.08 | 7.52 | 0.11 | 7.97 | 0.14 |
| 61 | 1.40 | 0.05 | 1.72 | 0.12 | 1.91 | 0.11 | 2.44 | 0.04 |
| 62 | 6.65 | 0.03 | 6.75 | 0.05 | 6.93 | 0.04 | 7.15 | 0.03 |
| 63 | 0.90 | 0.06 | 1.12 | 0.06 | 1.70 | 0.06 | 2.90 | 0.08 |
| 64 | 1.69 | 0.17 | 2.20 | 0.04 | 3.30 | 0.07 | 4.80 | 0.05 |
| 65 | 1.85 | 0.65 | 2.43 | 0.53 | 3.79 | 0.57 | 5.42 | 0.35 |
| 66 | 2.04 | 0.51 | 2.64 | 0.55 | 4.15 | 0.34 | 5.75 | 0.17 |
| 67 | 1.16 | 0.26 | 1.24 | 0.24 | 1.53 | 0.20 | 2.28 | 0.11 |
| 68 | 1.28 | 0.06 | 1.70 | 0.08 | 2.77 | 0.04 | 4.15 | 0.08 |
| 69 | 1.75 | 0.37 | 2.41 | 0.42 | 3.66 | 0.41 | 5.26 | 0.14 |
| 70 | 1.03 | 0.04 | 1.46 | 0.08 | 2.19 | 0.02 | 2.66 | 0.06 |
| 71 | 1.51 | 0.68 | 2.06 | 0.81 | 2.85 | 0.71 | 3.61 | 0.67 |
| 72 | 5.13 | 0.09 | 6.48 | 0.18 | 7.99 | 0.19 | 8.79 | 0.31 |
| 73 | 7.38 | 0.22 | 9.82 | 0.23 | 11.84 | 0.21 | 13.24 | 0.16 |
| 74 | 0.98 | 0.12 | 1.42 | 0.12 | 2.07 | 0.13 | 2.50 | 0.12 |
| 75 | 3.59 | 0.47 | 3.97 | 0.49 | 4.58 | 0.49 | 4.60 | 0.56 |
| 76 | 5.00 | 0.09 | 6.42 | 0.04 | 7.40 | 0.03 | 8.29 | 0.05 |
| 77 | 1.73 | 0.08 | 2.13 | 0.06 | 2.52 | 0.10 | 2.91 | 0.09 |
| 78 | 3.86 | 0.07 | 4.73 | 0.09 | 5.07 | 0.19 | 5.40 | 0.26 |
| 79 | 1.56 | 0.70 | 2.03 | 0.64 | 2.08 | 0.65 | 2.46 | 0.75 |
| 80 | 1.64 | 0.17 | 2.06 | 0.18 | 3.39 | 0.17 | 5.56 | 0.23 |
| 81 | 1.71 | 0.03 | 2.03 | 0.02 | 2.97 | 0.08 | 4.53 | 0.08 |
| 82 | 1.80 | 0.18 | 2.25 | 0.14 | 3.37 | 0.16 | 5.10 | 0.31 |
| 83 | 1.05 | 0.03 | 1.95 | 0.03 | 3.14 | 0.02 | 4.78 | 0.03 |
| 84 | 0.98 | 0.03 | 1.78 | 0.01 | 2.82 | 0.02 | 4.32 | 0.05 |
| 85 | 0.83 | 0.06 | 1.47 | 0.11 | 2.36 | 0.10 | 3.66 | 0.15 |
